# Strong sustained type I IFN signaling acts cell intrinsically to impair IFNγ responses and cause tuberculosis susceptibility

**DOI:** 10.1016/j.chom.2026.07.014

**Published:** 2026-08-14

**Authors:** Stefan A. Fattinger, Bianca Parisi, Roberto A. Chavez, Marian R. Fairgrieve, Ophelia V. Lee, Kristen C. Witt, Jesse J. Rodriguez, Elizabeth A. Turcotte, Ella C. Brydon, Harmandeep Dhaliwal, Angus Y. Lee, Dmitri I. Kotov, Russell E. Vance

**Affiliations:** 1 Division of Immunology and Molecular Medicine, Department of Molecular and Cell Biology, University of California, Berkeley, CA, USA.; 2 Cancer Research Laboratory, University of California, Berkeley, CA, USA.; 3 Center for Emerging and Neglected Diseases, University of California, Berkeley, CA, USA; 4 Present address: Division of Infectious Diseases, Department of Medicine, Andrew M. and Jane M. Bursky Center for Human Immunology and Immunotherapy Programs, Washington University School of Medicine, St. Louis, MO, 63110, USA.; 5 Howard Hughes Medical Institute, University of California, Berkeley, CA, USA.; 6 Lead contact

## Abstract

*Mycobacterium tuberculosis* (*Mtb*) causes over one million deaths annually, but most infected individuals never exhibit symptoms. Type I interferons (IFNs) have emerged as a major factor driving susceptibility to *Mtb*, but how type I IFNs impair immunity to *Mtb* is a key unresolved question. Here we show that an early effect of type I IFN during *Mtb* infection is the cell-intrinsic impairment of IFNγ signaling. IFNγ signaling is selectively impaired in the subset of infected macrophages experiencing high and sustained levels of type I IFN signaling. Genetic elimination of RESIST, a recently described positive regulator of type I IFN production, specifically eliminates the high and sustained type I IFN response, fully restores IFNγ signaling, and rescues susceptibility to *Mtb* without affecting basal type I IFN responses. Our results demonstrate that strong and sustained type I IFN responses specifically and cell intrinsically impair responsiveness to IFNγ to cause susceptibility to *Mtb*.

## eTOC Blurb

Fattinger et al. reveal how an antiviral immune response impairs antibacterial immunity and causes tuberculosis susceptibility. Upon *Mycobacterium tuberculosis* (*Mtb*) infection, strong sustained type I IFN signaling cell intrinsically suppresses IFNγ responsiveness, rendering macrophages permissive to bacterial growth. The results illustrate how *Mtb* exploits host immune pathways to promote pathogenesis.

## Graphical Abstract

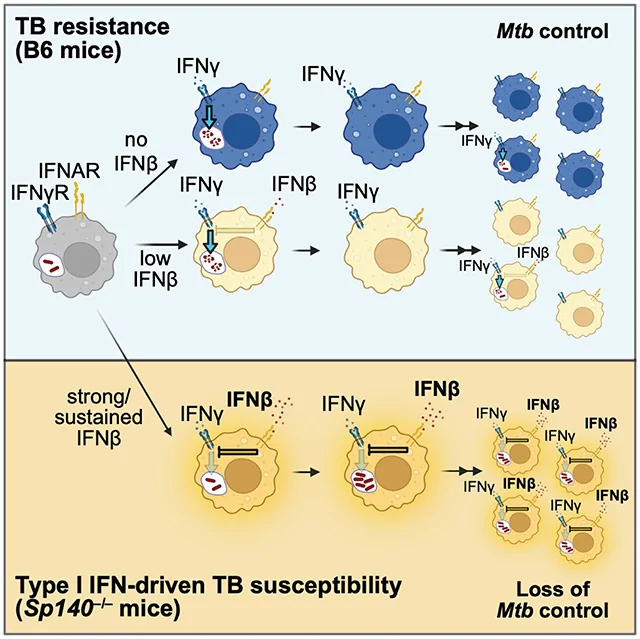

## INTRODUCTION

Tuberculosis (TB) is caused by *Mycobacterium tuberculosis* (*Mtb*) and is the deadliest infectious disease of humans, resulting in over one million deaths annually^1^. Standard TB treatment requires a 4-6 month antibiotic course that is increasingly ineffective against multi-drug resistant *Mtb*. *Mtb* infection elicits a robust innate and adaptive immune response that protects most but not all infected individuals. A major unresolved question is why immunity fails to prevent disease in some individuals.

Interferon-γ (IFNγ) is implicated in protection against mycobacterial infections in humans^2–5^, and is essential in mice for resistance to *Mtb*^6–10^. However, most susceptible humans and mice robustly produce IFNγ in response to *Mtb*, indicating that IFNγ production is often insufficient for protection. Indeed, BCG and a recent vaccine candidate fail to protect adults from infection, despite eliciting IFNγ-producing T cells^11–15^. In contrast to IFNγ, type I IFNs (e.g., IFNβ, IFNα) are clearly associated with *Mtb* disease progression in mice and humans^16–30^. Type I IFNs impair IL-1-mediated immunity to *Mtb* via mechanisms including lipid mediator production or upregulation of IL-1 receptor antagonist (IL-1Ra) or IL-10^25,29,31^. However, type I IFN signaling can also promote susceptibility independent of IL-1 signaling^25^, indicating that additional mechanisms may contribute to type I IFN–driven susceptibility^32,33^.

Recently, we identified SP140 as a key negative regulator of type I IFN responses^34,35^. Loss of SP140 increases type I IFN production, causing uncontrolled *Mtb* replication characterized by hypoxic granulomas, and an exacerbated inflammatory phenotype resembling human clinical TB^27,34,36^. The enhanced susceptibility of *Sp140*^−/−^ mice is rescued by crossing to *Ifnar*^−/−^ mice^27,34^, establishing *Sp140*^−/−^ mice as a model of type I IFN-driven TB. *Ifnar*-deficiency does not greatly affect susceptibility of wild-type B6 mice to *Mtb*, implying that basal type I IFN levels in B6 mice are not pathogenic during *Mtb* infection^29,31,37–39^. It remains unclear how elevated, but not basal, type I IFN specifically impairs immunity to *Mtb*.

SP140 represses two tandemly duplicated genes, *Resist1* and *Resist2*, which encode the identical protein RESIST (REgulated Stimulator of Interferon via Stabilization of Transcript)^35^. *Sp140*^−/−^ macrophages express RESIST, which stabilizes *Ifnb1* mRNA by impairing CCR4-NOT polyA-tail deadenylase activity, thereby limiting *Ifnb1* mRNA turnover^35^. *Resist1/2*-deficiency eliminates the elevated *Ifnb1* mRNA and IFNβ protein in *Sp140*^−/−^ mice, restoring basal B6-like IFNβ expression^35^, but whether this also restores *Mtb* resistance remains unknown.

In the lungs of humans and mice, intracellular *Mtb* replication occurs mainly within a heterogenous population of CD64^+^ macrophages, including interstitial macrophages (IMs), a type of myeloid cell in which T cells only inconsistently mediate *Mtb* control^40,41^. Some *Mtb* replication may also occur in neutrophils^42,43^. Cell-type specific deletion of *Ifnar* in CD64^+^ myeloid cells rescues *Sp140*^−/−^ susceptibility^27^, implying that type I IFNs act on myeloid cells, but whether they act cell intrinsically on infected macrophages to impair *Mtb* control, or cell extrinsically—e.g., by inducing macrophage IL-10 production to suppress other cells—remains unclear. We hypothesized that inconsistent IFNγ-mediated control of *Mtb* might arise from differential macrophage exposure to type I IFNs. Supporting this, type I IFN signaling can impair induction of certain IFNγ-induced interferon stimulated genes (ISGs)^44–47^, and elevated type I IFN ISG induction correlates with decreased IFNγ-induced ISG expression and worse outcomes in human *Mycobacterium leprae* infections^48^. Type I IFNs also impair IFNγ signaling in *Listeria*-infected mice^49^. During *Mtb* infection, type I IFN can also impair IL-12 expression—a key inducer of IFNγ—cell extrinsically via IL-10^50^. *In vivo*, we recently found that the enhanced type I IFN response in *Sp140*^−/−^ mice correlates with an attenuated IFNγ response in *Mtb*-infected interstitial macrophages^27^, but a causal relationship was not established. In addition, analyses were performed at day 25 post infection, when bacterial loads had already diverged between susceptible and resistant mice, confounding cause and effect, and there was no evidence that cells with decreased IFNγ responsiveness were a susceptible niche for *Mtb*. Thus, it remains unclear whether type I IFN signaling cell intrinsically impairs the IFNγ response of infected macrophages *in vivo*, and whether such impairment causes susceptibility to *Mtb*.

Here, we use *Sp140*^−/−^ mice to systematically dissect the emergence and cause of type I IFN-driven susceptibility to *Mtb*. We show that strong and sustained type I IFN signaling selectively impairs IFNγ responses in a subset of IMs *in vivo*, and that this subset harbors the highest *Mtb* burdens. We further uncover a temporal progression in which type I IFN signaling precedes and then diminishes IFNγ responsiveness, rendering IMs a permissive niche for *Mtb*. Finally, genetic elimination of RESIST in *Sp140*^−/−^ mice demonstrates that sustained and strong (but not basal) type I IFN signaling specifically suppresses IFNγ responsiveness and drives *Mtb* susceptibility.

## RESULTS

### IFNAR-STAT2-IRF9 signaling broadly impairs IFNγ signaling

**Figure 1.**
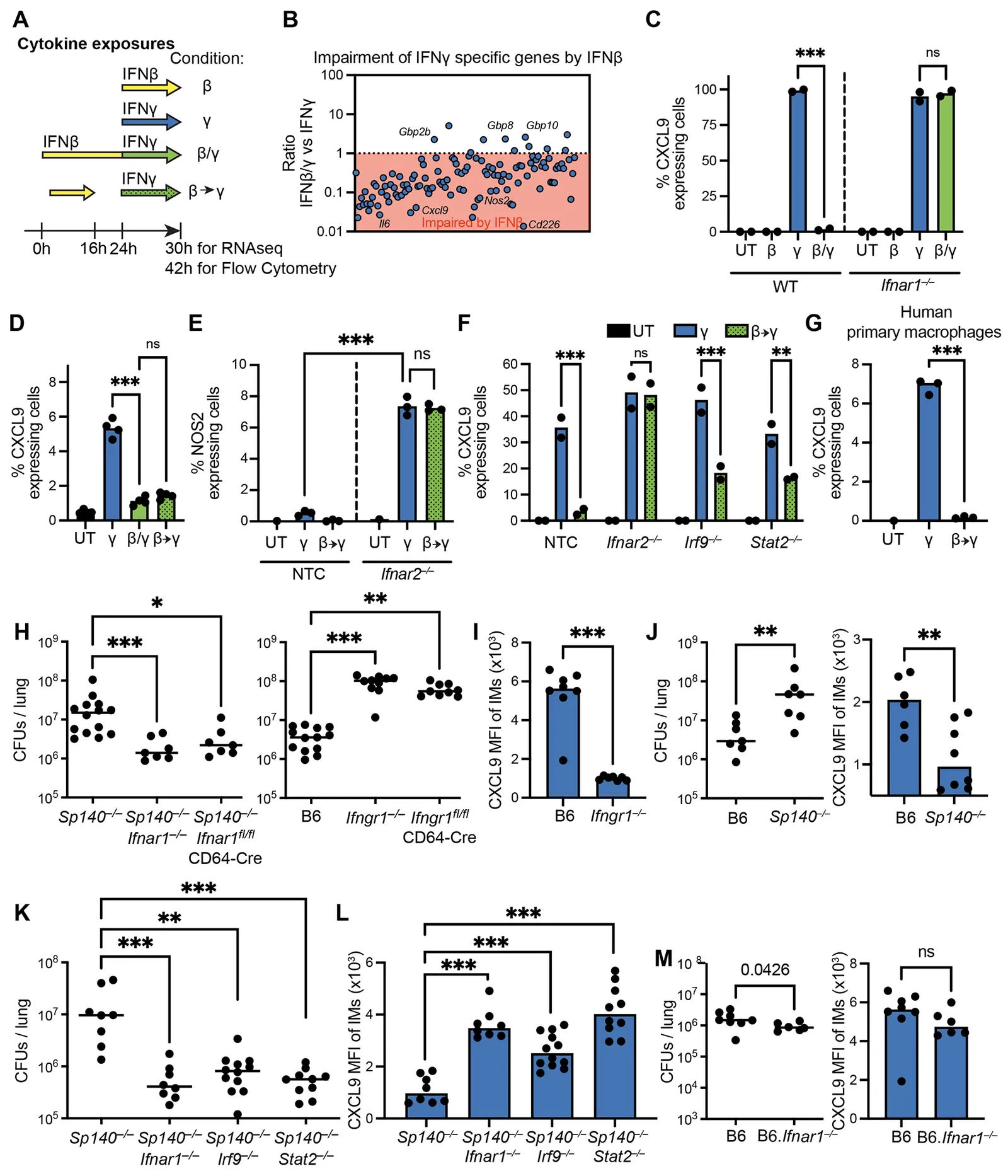
IFNAR-STAT2-IRF9 signaling broadly impairs IFNγ signaling (**A**) Cytokine exposures for RNAseq and flow cytometry analysis. (**B**) Ratio of IFNγ-specific gene induction in BMMs upon IFNβ/γ vs IFNγ treatment. Average of *n* = 3 is plotted in (B). (**C**) CXCL9 protein expression by BMMs from B6 wild-type (WT) or *Ifnar1*^−/−^ mice upon exposure to IFNβ, IFNγ or IFNβ/γ. UT, Untreated (**D**) CXCL9 expression by BMMs, comparing IFNβ pre-exposure without (β/γ) or with (β→γ) its removal prior to IFNγ stimulation. (**E**) NOS2 expression by CRISPR-edited BMMs. NTC, Non-Target Control. (**F**) CXCL9-expression by CRISPR-edited BMMs. NTC, Non-Target Control. (**G**) CXCL9 expression by human primary macrophages. (**H**) Colony Forming Units (CFUs) per lung from *Mtb*-infected B6 or type I IFN susceptible *Sp140*^−/−^ mice with global or CD64^+^ macrophage-specific deletion of *Ifnar1* or *Ifngr1* at day 25 post infection (pi) (**I**) Mean Fluorescence Intensity (MFI) of CXCL9 staining of interstitial macrophages (IMs) from *Mtb*-infected B6 and *Ifngr1*^−/−^ mice at day 25 pi. (**J**) CFUs per lung and MFI of CXCL9 staining in IMs from *Mtb*-infected B6 and *Sp140*^−/−^ mice at day 25 pi. (**K**) CFUs per lung and (**L**) MFI of CXCL9 staining of IMs from indicated mouse strains at day 28-29 pi. (**M**) CFUs per lung and MFI of CXCL9 staining in IMs from *Mtb*-infected B6 and B6.*Ifnar1*^−/−^ mice at day 25-26 pi. (C-G) Representative data from ≥ 3 biological replica with n ≥ 2 per condition. Statistical significance was calculated in (C,E,F) with two-way ANOVA with (C,E) Sidak’s multiple comparison test or (F) Turkey’s multiple comparison test; in (D,G) one-way ANOVA with Sidak’s multiple comparison test. (H-M) Data was combined from ≥ 2 biological replica with n ≥ 6 per group. Statistical significance was calculated for CFU data in (H, K) with Kruskal-Wallis test with Dunn’s multiple comparison test in (J, M) with Mann-Whitney test and, for MFI data in (I,J,M) Welch’s t-test and in (L) Brown-Forsythe/Welch ANOVA test with Dunnett’s T3 multiple comparisons test. *p < 0.05, **p < 0.01, ***p < 0.001, ns = not significant. See also figure S1 and figure S2.

To test whether type I IFN globally impairs IFNγ-induced transcriptional responses, or just certain IFNγ-induced genes^44–47^, we performed RNAseq on mouse bone marrow-derived macrophages (BMMs) exposed to IFNβ (type I IFN) alone, IFNγ alone, or both simultaneously (conditions β, γ and β/γ; Figure 1A). Most ISGs are similarly induced by IFNβ and IFNγ, but we previously defined 21 “IFNγ signature genes” preferentially induced by IFNγ and upregulated during *Mtb* infection^27^. IFNβ pre-exposure blunted IFNγ-induced expression of almost all IFNγ signature genes (Figure S1A), with the notable exception of *Gbp8*, which was induced by IFNγ similarly in IFNβ-pretreated and control cells. To test if type I IFN impairs the IFNγ response beyond the signature genes, we identified 121 ISGs induced at least two-fold more by IFNγ than IFNβ (Figure S1B). Strikingly, 91% (110/121) of these genes were suppressed by IFNβ pre-exposure (Figure 1B). *Gbp2b*, *Gbp8* and *Gbp10* were among the 11 ISGs (Figure S1C) induced by IFNγ despite IFNβ pretreatment.

The chemokine CXCL9 is a canonical IFNγ-induced ISG that is highly expressed during human and non-human primate *Mtb* infections, and has been suggested to promote an effective T cell mediated immune response^51,52^. In our RNAseq dataset, *Cxcl9* was among the most IFNγ-upregulated genes, induced >8-fold more by IFNγ than IFNβ (Figure S1B), a result validated by RT-qPCR (Figure S1D). Despite some induction of *Cxcl9* transcripts by IFNβ (Figures S1B, S1D), intracellular staining and flow cytometry showed that IFNβ pre-exposure fully abolished CXCL9 protein induction by IFNγ (Figures 1C, S1E). Exposure to IFNβ, IFNγ, or both cytokines did not impact cell viability (Figure S1F), and IFNβ suppression of CXCL9 induction required IFNAR (Figures 1C, S1E). A 10-fold increase in IFNγ concentration could not overcome IFNβ-mediated suppression, though co-stimulation with TNF or TLR-agonists partially reversed the inhibition in an IFNβ concentration-dependent manner (Figure S1H). Impairment of IFNγ signaling persisted after IFNβ removal, even after *Rsad2* transcripts (encoding Viperin) had returned to baseline (Figures 1D, S1I, compare β/γ to β→γ, see Figure 1A for exposure details). Indeed, a time course analysis revealed that impairment can last up to 32h upon IFNβ removal (Figure S1J).

Nitric Oxide Synthase 2 (NOS2) is another IFNγ-induced ISG implicated in *Mtb* protection in mice^8,53–55^. In our RNAseq dataset, *Nos2* was highly and specifically induced by IFNγ (Figure S1B). In sharp contrast to CXCL9, NOS2 was barely detectable at the protein level in IFNγ-treated cells (Figure 1E), consistent with previous reports^56^. Strikingly, however, *Ifnar2*^−/−^ BMMs generated by CRISPR induced abundant NOS2 protein in response to IFNγ (Figure 1E), implying that tonic type I IFN signaling is sufficient to impair IFNγ-induced NOS2 expression and explaining the previously observed failure of IFNγ to induce NOS2 in wild-type cells^56^. However, unlike CXCL9, NOS2 protein expression was not induced exclusively by IFNγ, and TLR agonists also fully reversed its suppression by IFNβ (Figure S1K). Given these observations, and the fact that the role of NOS2 in human TB protection remains debated^57^, we chose CXCL9 as the most reliable and specific marker for the IFNγ response in subsequent analyses.

Signaling downstream of IFNAR involves STAT1, STAT2 and IRF9, believed to form a complex (ISGF3) that binds interferon-sensitive response elements to induce type I ISG expression^58^. To test whether these transducers mediate the impairment of IFNγ responsiveness, we generated STAT2- and IRF9-deficient BMMs and exposed them to IFNγ ± IFNβ pre-exposure. Both STAT2- and IRF9-deficiency partially prevented type I IFN-driven inhibition (Figure 1F).

To confirm relevance to human cells, we exposed human THP-1 cells and primary monocyte-derived macrophages to IFNs. In both cell types, IFNβ robustly inhibited IFNγ-induced CXCL9 induction, demonstrating that type I IFN also impairs the IFNγ response in human macrophages (Figures 1G, S1L). Together, these data establish CXCL9 as a marker for IFNγ signaling and, with prior observations^44–47^, demonstrate that in mouse and human macrophages, type I IFN signaling downstream of IFNAR engages STAT2 and IRF9 to robustly and sustainably impair IFNγ responsiveness.

### Type I IFNs impair the IFNγ response to *Mtb in vivo*

*Sp140*^−/−^ mice express elevated type I IFN and are a robust model of type I IFN-driven *Mtb* susceptibility^34^. We infected *Sp140*^−/−^, *Sp140^−/−^Ifnar1*^−/−^ and *Sp140^−/−^Ifnar1*^fl/fl^CD64-Cre mice with a low aerosolized dose (20-100 colony forming units (CFUs)) of *Mtb* Erdman strain expressing a fluorescent reporter validated to correlate with *Mtb* CFU *in vivo*^27^. *Ifnar* deletion in CD64^+^ cells rescued *Mtb* susceptibility indistinguishably from global *Ifnar* deletion, confirming that type I IFNs act on CD64^+^ myeloid cells to cause susceptibility^27^ (Figure 1H). To determine if CD64^+^ myeloid cells are also the main cell type through which IFNγ controls *Mtb* replication, we generated *Ifngr1*^fl/fl^CD64-Cre mice, which specifically lack IFNGR on myeloid cells. Strikingly, these mice were as susceptible as global *Ifngr1*-deficient mice (Figure 1H), suggesting that CD64^+^ myeloid cells mediate both type I IFN-driven susceptibility and IFNγ-dependent protection. We therefore focused our analysis on CD64^+^ IMs, a major intracellular niche for *Mtb* replication^41^ (see Figure S2A for gating strategy).

Based on our *in vitro* results (Figures 1A–G, S1), we tested whether CXCL9 is a reliable marker of IFNγ responsiveness in infected IMs *in vivo*. At day 25 post infection (pi), intracellular CXCL9 was negligible in neutrophils (Figures S2B–C), while IMs from wild-type B6 mice expressed significantly higher CXCL9 than *Ifngr1*^−/−^ mice (Figures 1I, S2B–C). Differences in autofluorescence across genotypes were excluded as a confounding factor (Figures S2D–E). Thus, we selected CXCL9 as a reliable *in vivo* marker of IFNγ responsiveness during *Mtb* infection.

Next, to test whether enhanced type I IFN signaling represses IFNγ responsiveness *in vivo*, we infected B6 and *Sp140*-deficient mice. IMs from *Sp140*^−/−^ mice expressed less CXCL9 than B6 mice, indicative of reduced IFNγ responsiveness (Figures 1J, S2D–E). To test whether this decrease was due to IFNAR signaling, we examined *Sp140^−/−^Ifnar1*^−/−^ mice. As reported previously^34^, IFNAR-deficiency rescued *Mtb* susceptibility in *Sp140*^−/−^ mice (Figure 1K). We also found IFNAR-deficiency rescued IFNγ signaling in IMs, as read out by CXCL9 expression, compared to *Sp140*^−/−^ mice (Figure 1L). Notably, *Sp140*^−/−^ mice harboring single *Stat2* or *Irf9* null mutations (generated by CRISPR) were also rescued for both type I IFN-driven susceptibility and IFNγ responsiveness (Figures 1K, L). To validate these results, we also examined NOS2 expression: compared to CXCL9, NOS2 was mainly expressed in infected (versus uninfected bystander) IMs (Figures S2F–G), and focusing on infected IMs, we observed suppression of NOS2 expression in cells with intact type I IFN signaling (Figures S2H–I).

During *Mtb* infection, B6 mice express lower type I IFN than *Sp140*^−/−^ mice, and do not show consistent type I IFN-driven susceptibility to *Mtb*^29,31,37–39^. Thus, if *Sp140*^−/−^ susceptibility is due to type I IFN impairment of the IFNγ response, we should not observe such impairment in wild-type B6 mice. Indeed, *Ifnar1*-deficiency had no effect on CXCL9 or NOS2 expression on a B6 background (Figures 1M, S2J). If anything, we detected lower CXCL9 levels in B6.*Ifnar1*^−/−^ than B6 IFNAR-sufficient mice.

Overall, these findings indicate that elevated and/or sustained type I IFN in *Sp140*^−/−^ mice impairs the IFNγ response to *Mtb in vivo*, and that disrupting type I IFN signaling via STAT2 or IRF9 deficiency is sufficient to overcome this effect.

### Type I IFNs impair IFNγ-responsiveness and *Mtb* control cell intrinsically

**Figure 2.**
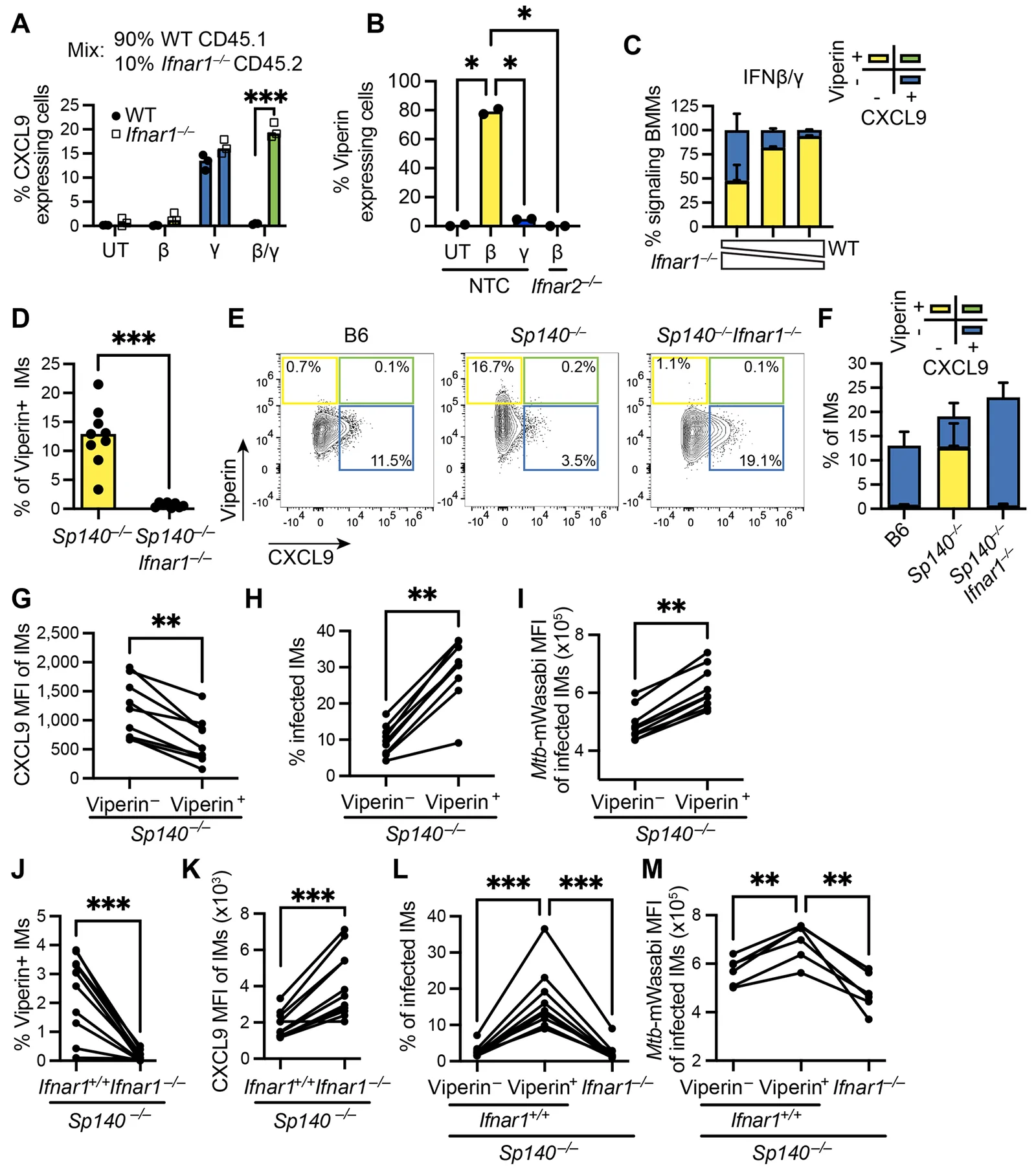
Type I IFNs impair IFNγ-responsiveness and *Mtb* control cell intrinsically (**A**) Quantification of CXCL9 expression in WT CD45.1 and *Ifnar1*^−/−^ CD45.2 BMMs from a mixed culture with an overabundance of WT CD45.1 cells (9:1) upon exposure to IFNβ, IFNγ and both (IFNβ/γ). (**B**) Viperin-expression by CRISPR-edited BMMs upon IFNβ or IFNγ. NTC, Non-Target Control; UT, Untreated. (**C**) Quantification of CXCL9 and/or Viperin expressing cells from mixed WT : *Ifnar1*^−/−^ BMM cultures at various ratios upon exposure to both IFNβ and IFNγ (IFNβ/γ). (**D**) Percent Viperin expressing IMs from *Mtb*-infected *Sp140*^−/−^ and *Sp140*^−/−^*Ifnar1*^−/−^ mice at day 28-29 pi. (**E**) Representative flow cytometry plots and (**F**) analysis of IMs for CXCL9 and Viperin expression from *Mtb*-infected B6, *Sp140*^−/−^ and *Sp140*^−/−^*Ifnar1*^−/−^ mice at day 28-29 pi. (**G**) CXCL9 MFI, (**H**) percent *Mtb* infection, and (**I**) *Mtb*-mWasabi MFI in Viperin-positive and -negative IMs from *Mtb*-infected *Sp140*^−/−^ mice at day 28-29 pi. (**J-M**) *Mtb*-infected mixed *Sp140*^−/−^ bone marrow chimeras containing IFNAR-proficient (CD45.1) and -deficient cells (CD45.2) at day 25-26 pi analyzed for (**J**) percent Viperin positive IMs, (**K**) CXCL9 MFI, (**L**) percent *Mtb* infection, and (**M**) MFI of *Mtb*-mWasabi among Viperin/IFNAR-expressing IM subsets. (C, F) Data are represented as mean ± SD. (A-C) Representative data from ≥ 3 biological replica with n ≥ 2 per condition. (D, F-M) Data was combined from ≥ 2 biological replica with n ≥ 7 per group. Statistical significance was calculated (A-B) with Two-way ANOVA and Sidak’s multiple comparison test, in (D) Welch’s t-test, in (G-K) Paired t-test, in (L-M) RM one-way ANOVA with Sidak’s multiple comparison test. *p < 0.05, **p < 0.01, ***p < 0.001, ns = not significant. See also figure S3.

Type I IFN signaling has been suggested to impair the IFNγ response cell intrinsically by downregulating IFNγ receptor levels, or cell extrinsically via the immunosuppressive cytokine IL-10^47–50^. To distinguish these possibilities, we mixed WT CD45.1 and *Ifnar1*^−/−^ CD45.2 BMMs at a 9:1 ratio and exposed them to IFNβ and IFNγ (IFNβ/γ) *in vitro*. IFNβ impaired the IFNγ response in WT cells, but *Ifnar1*^−/−^ cells in the same culture robustly induced CXCL9, indicating that IFNβ-responsive cells do not suppress neighboring non-responsive cells (Figure 2A).

Next, we sought to identify an ISG marking type I IFN–responsive cells by flow cytometry, and found that *Rsad2* (encoding Viperin) was abundantly expressed upon IFNβ exposure at the mRNA (Figure S1I) and protein levels (Figure 2B). Viperin protein expression was specific to type I IFN signaling (not observed upon IFNγ exposure) and IFNAR-dependent (Figure 2B). In mixed WT and *Ifnar1*^−/−^ BMM cultures exposed to IFNβ/γ, CXCL9 and Viperin expression were mutually exclusive, further supporting a cell-intrinsic mechanism for the type I IFN-dependent impairment of IFNγ responses (Figure 2C). Together, these data indicate that, *in vitro*, type I IFN acts cell intrinsically and does not induce a soluble mediator sufficient to suppress IFNγ signaling.

We then examined Viperin and CXCL9 expression by IMs in *Mtb*-infected mice. As expected, Viperin staining was IFNAR-dependent in *Mtb*-infected *Sp140*^−/−^ mice (Figure 2D) and unaffected by autofluorescence (Figures S3A–B). Consistent with the *in vitro* results, CXCL9 and Viperin expression in IMs was mutually exclusive (Figures 2E–F), as expected if type I IFN-responsive cells fail to respond to IFNγ. Moreover, in *Sp140*^−/−^ mice, Viperin-positive IMs expressed reduced CXCL9, and were more frequently infected with elevated *Mtb* burdens (Figures 2G–I), suggesting a direct connection between elevated type I IFN signaling, reduced IFNγ-responsiveness, and elevated *Mtb* burdens within the same cell. However, a caveat is that these results could be confounded by the different *Mtb* burdens in *Sp140*^−/−^ versus *Sp140^−/−^Ifnar1*^−/−^ mice.

To control for differing bacterial burdens between *Sp140*^−/−^ and *Sp140^−/−^Ifnar1*^−/−^ mice, we established mixed bone marrow chimeras in which CD45.1 IFNAR-proficient and CD45.2 IFNAR-deficient *Sp140*^−/−^ bone marrow cells were used to reconstitute *Sp140*^−/−^ recipients, alongside control chimeras reconstituted with IFNAR-proficient CD45.1 and CD45.2 cells (Figures S3C–F). Mice were infected with *Mtb* >8 weeks after reconstitution. In these chimeras, IFNAR-proficient and -deficient cells respond to *Mtb* and IFNγ within the same inflammatory environment. Consistent with the results above, Viperin expression was IFNAR-dependent and CXCL9 expression was restored in *Ifnar1*^−/−^ IMs (Figures 2J–K). Notably, Viperin staining revealed that only a minor fraction (1-4%) of *Ifnar1*^+/+^ IMs actively respond to type I IFN (Figure 2J, control in Figure S3C). Because Viperin induction is transient (Figure S1I), it may fail to identify all cells that have sensed type I IFNs (see below). Nevertheless, we consistently found decreased CXCL9 levels (Figure S3G) and elevated *Mtb* burdens in the few Viperin-expressing *Ifnar1^+/+^* IMs compared to Viperin-negative *Ifnar1*^+/+^ or *Ifnar1*^−/−^ IMs (Figures 2L–M). Although some IFNAR^+^ but Viperin-negative cells may never have been exposed to IFNβ, we could still detect increased CXCL9 expression and decreased *Mtb* infection by percentage and MFI in the total *Ifnar1*^+/+^ population (Figures S3D–F).

In summary, these observations suggest that in IMs, type I IFN predominantly impairs IFNγ response in a cell-intrinsic manner to promote susceptibility to *Mtb*.

### Sustained and strong type I IFN–signaling impairs IFNγ responsiveness

**Figure 3.**
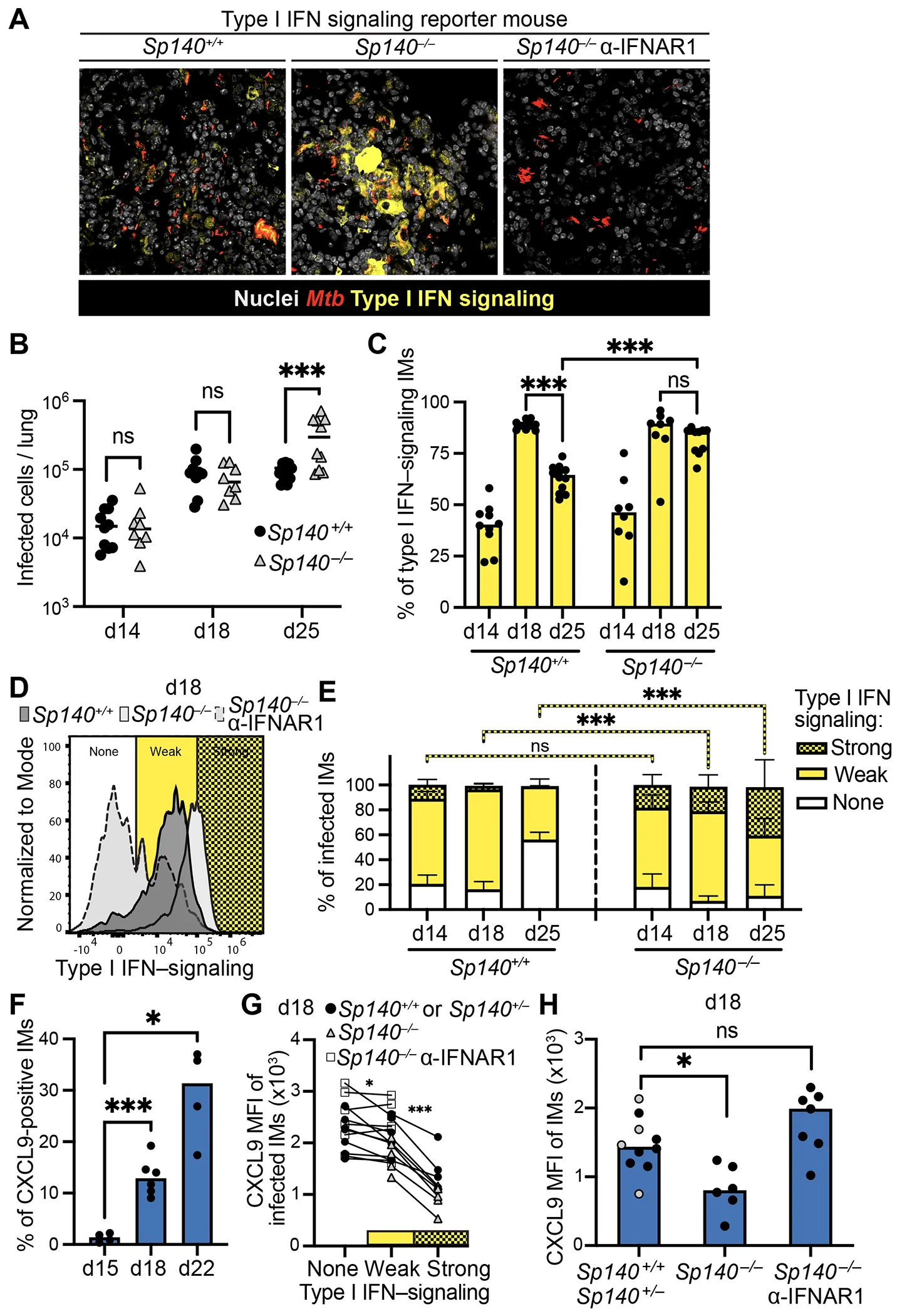
Sustained and strong type I IFN–signaling impairs the response to IFNγ and precedes susceptibility to *Mtb* (**A**) Representative micrograph of lung tissue from *Mtb*-infected *Sp140*^+/+^ and *Sp140*^−/−^ Mx1-GFP reporter mice ± administration of anti-IFNAR1 antibodies at day 18 pi. (**B**) Quantification of *Mtb*-infected cells and (**C**) Mx1-GFP-expression on IMs from lung tissue of *Sp140*^+/+^ and *Sp140*^−/−^ mice at day 14, 18 and 25 pi. (**D**) Representative flow cytometry histogram of Mx1-GFP expression of infected IMs from lung tissue of *Sp140*^+/+^ and *Sp140*^−/−^ mice without or with administration of anti-IFNAR1 antibodies at day 18 pi. “Weak” expression is defined as a level seen in B6 mice that is greater than background, whereas strong expression is the level seen in less than 5% of cells in infected B6 mice at day 18 pi (but by many more cells in *Sp140*^−/−^ mice). (**E**) Quantification of percent infected IMs with different levels of type I IFN signaling (as defined in (D)) from lung tissue of *Sp140*^+/+^ and *Sp140*^−/−^ mice at day 14, 18 and 25 pi. Data are represented as mean ± SD. (**F**) Percent IMs that are CXCL9^+^ from lung tissue of *Mtb*-infected B6 mice at day 15, 18 and 22 pi. (**G**) Comparison of CXCL9 MFI between none, weak and strong type I IFN signaling IMs (as defined in (D)) from lung tissue of *Sp140*^+/+^ *or Sp140*^+/−^ and *Sp140*^−/−^ mice without or with administration of anti-IFNAR1 antibodies at day 18 pi. (**H**) CXCL9 MFI of IMs from lung tissue of *Sp140*^+/+^ (black dots) *or Sp140*^+/−^ (gray dots) and *Sp140*^−/−^ mice without or with administration of anti-IFNAR1 antibodies at day 18 pi. (B-C, E-H) Data was combined from ≥ 2 biological replica with n ≥ 4 per group. Statistical significance was calculated in (B, C, E) with Two-way ANOVA and Sidak’s multiple comparison test, in (F, H) with Brown-Forsythe and Welch ANOVA test and Dunnett’s T3 multiple comparisons test, in (G) Mixed-effects analysis, with the Geisser-Greenhouse correction and with Sidak’s multiple comparison test with individual variances computed for each comparison. *p < 0.05, **p < 0.01, ***p < 0.001, ns = not significant. See also figure S4.

Although Viperin expression marked some type I IFN-responsive cells, Viperin expression is transient and lost before the repressive effects of type I IFN on IFNγ signaling resolve (Figures 1D, S1I), so it likely underreports prior type I IFN exposure. As a more sensitive reporter, we used a previously described type I IFN signaling reporter mouse^59^, in which GFP is expressed under the native promoter of *Mx1*, a type I IFN-induced gene transcribed but not translated into functional protein in mice. The long half-life of GFP allows sensitive, sustained detection of type I IFN-responsive cells. We confirmed in BMMs that GFP is induced upon IFNβ (Figure S4A). On day 18 post-*Mtb* infection, we detected type I IFN-responsive cells in lung lesions of *Sp140*^−/−^Mx1-GFP mice (Figures 3A, S4B), with much weaker reporter expression in B6.Mx1-GFP mice. As a key control, anti-IFNAR1 antibody, previously shown to rescue *Sp140*^−/−^ susceptibility^34^, reduced Mx1-GFP expression below B6 levels, indicating that the reporter is specific and sensitive for type I IFN signaling *in vivo* in *Mtb*-infected mice (Figures 3A, S4B).

We analyzed the lungs of B6 and *Sp140*^−/−^Mx1-GFP reporter mice at days 14, 18 and 25 pi. While type I IFN signaling was detected in IMs as early as day 14 in both genotypes, the enhanced susceptibility of *Sp140*^−/−^ mice only became apparent at day 25 pi (Figures 3B–C). Surprisingly, at days 14 and 18 pi, the frequency of type I IFN-sensing IMs was similar across genotypes. However, by day 25 pi, the prevalence of GFP^+^ IMs decreased in B6 mice but was sustained in *Sp140*^−/−^ mice (Figure 3C), similar to a recently reported sustained type I IFN response in C3HeB/FeJ susceptible mice^60^. The reporter allowed us to distinguish three levels of type I IFN signaling—none, weak and strong (Figure 3D). We defined weak expression as the level seen in B6 mice that is greater than background, and strong expression as the level seen in <5% of infected B6 cells at day 18 pi (but in many more cells in *Sp140*^−/−^ mice). At day 14 pi, IFN signaling strength was similar between B6 and *Sp140*^−/−^ mice (Figure 3E). However, by day 18 pi, more IMs responded strongly to type I IFN in *Sp140*^−/−^ versus B6 mice (Figure 3E). While weak type I IFN signaling declined over time in both genotypes, strong signaling increased in *Sp140*^−/−^ mice by day 25 pi but largely disappeared in B6 mice (Figure 3E). These observations demonstrate that by day 18 pi, a substantial subset of IMs from *Sp140*^−/−^ mice exhibit a stronger, more sustained type I IFN response that precedes the appearance of elevated *Mtb* burdens.

In contrast to type I IFN signaling, the IFNγ response (assessed by CXCL9 expression) was only apparent starting at day 18 pi (Figure 3F), likely coinciding with the arrival of *Mtb*-specific T cells^61,62^. At this timepoint, cells mounting a strong type I IFN response showed markedly decreased CXCL9 expression compared to cells responding weakly or undetectably (Figure 3G). This effect was evident in all *Sp140*^−/−^ mice (5/5) and in the few B6 mice with detectable strong type I IFN signaling (4/9, Figure 3G). Because considerably more cells responded strongly to type I IFN in *Sp140*^−/−^ mice, these mice showed an overall decrease in lung IM CXCL9 levels compared to B6 (Figure 3H). At this timepoint (day 18 pi), CFU burdens were similar and CD4 T cells were equally recruited to *Mtb* within infected lesions of B6 and *Sp140*^−/−^ mice ± anti-IFNAR1 treatment (Figures S4C–E). However, by day ≥25 pi, CD4 T cells failed to localize to infected lesions in *Sp140*^−/−^ mice, while preferentially localizing there in B6 mice (Figures S4F–G). Thus, type I IFN-dependent defects in IFNγ signaling preceded loss of bacterial control and dysfunctional T cell localization.

Together, our results demonstrate that type I IFN signaling occurs at early timepoints during *Mtb* infection, preceding the onset of the IFNγ response. In B6 mice, the type I IFN response is weak and transient, so responsiveness to IFNγ at later timepoints is unimpaired, and bacterial restriction and T cell recruitment remain intact. In *Sp140*^−/−^ mice, however, many cells respond strongly and persistently to type I IFN and develop defective IFNγ responsiveness, resulting in uncontrolled *Mtb* replication and impaired T cell recruitment, which may further reduce the ability of IMs to restrict *Mtb* growth.

### RESIST promotes type I IFN responses and impairs IFNγ responses and *Mtb* control

**Figure 4.**
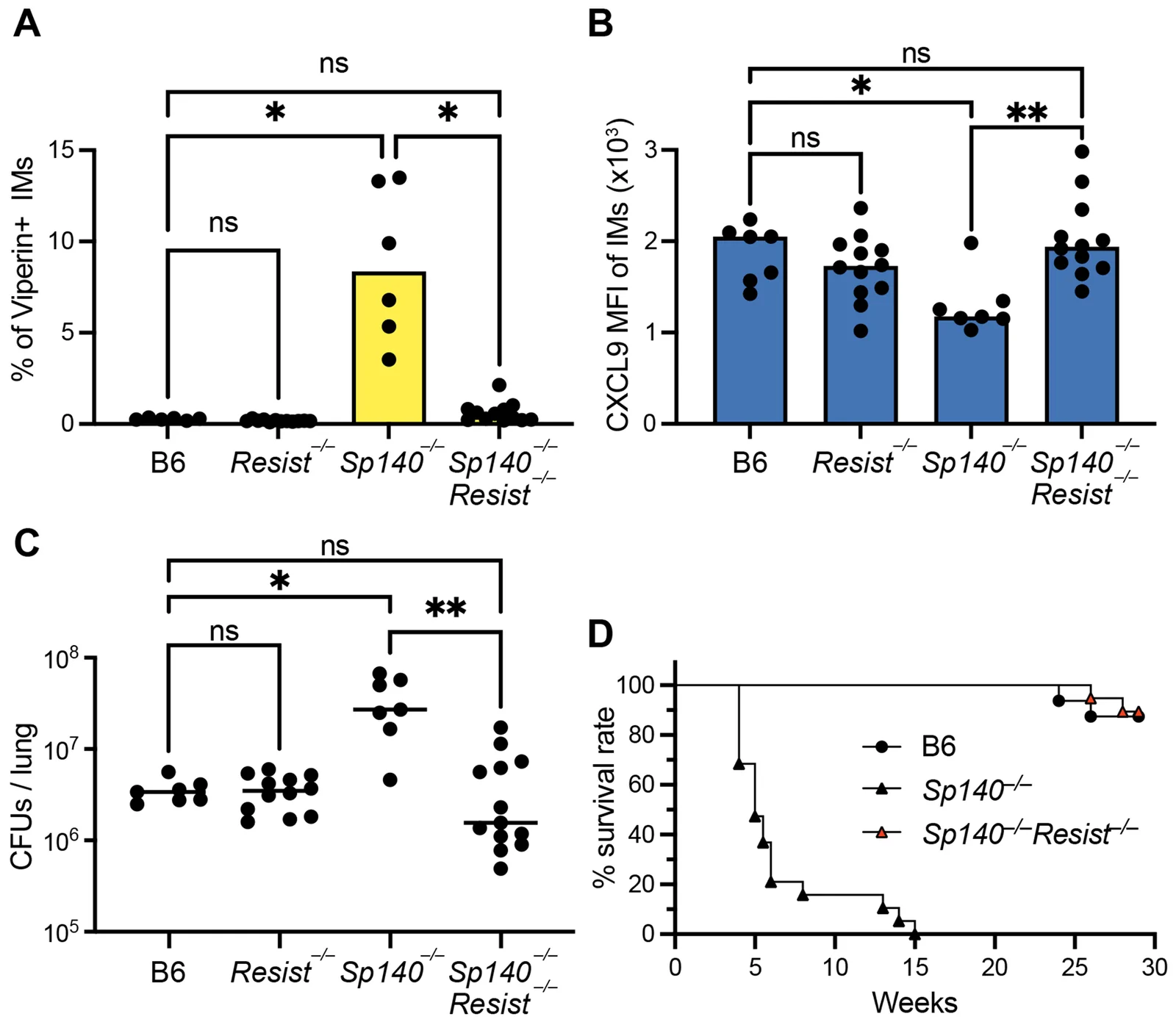
RESIST stabilizes and strengthens type I IFN responses, impairs IFNγ responses, and promotes susceptibility to *Mtb* (**A**) Percent Viperin expression, (**B**) CXCL9 MFI of IMs and (**C**) CFUs from lung tissue of B6, *Resist*^−/−^, *Sp140*^−/−^ and *Sp140*^−/−^*Resist*^−/−^ mice at day 26-28 pi. (**D**) Survival experiment. (A-D) Data was combined from ≥ 2 biological replica with n ≥ 6 per group. Statistical analysis was calculated in (A-B) with Brown-Forsythe/Welch ANOVA test with Dunnett’s T3 multiple comparisons test and in (C) with Kruskal-Wallis test and Dunn’s multiple comparison test. *p < 0.05, **p < 0.01, ***p < 0.001, ns = not significant. See also figure S5.

Genetic or antibody-mediated suppression of IFNAR signaling rescues IFNγ signaling and *Sp140*^−/−^ susceptibility to *Mtb*^34^ (Figures 1K, 3G–H). However, these approaches eliminate all type I IFN signaling and thus cannot specifically eliminate the sustained, strong type I IFN response that our data suggest drives susceptibility. We recently discovered that RESIST (encoded by *Resist1* and *Resist2*) is a direct target of SP140 repression and a crucial positive regulator required for enhanced (but not basal) IFNβ expression in *Sp140*^−/−^ mice^35^. To test whether sustained and strong type I IFN signaling causes type I IFN-driven *Mtb* susceptibility, we asked whether genetic loss of *Resist1* and *Resist2* (*Resist*^−/−^) rescues *Sp140*^−/−^ mice. Indeed, while *Resist*-deficiency had little impact in B6 mice (where *Resist* is not normally expressed), *Sp140^−/−^Resist*^−/−^ mice were fully rescued for appropriate type I IFN signaling compared to *Sp140*^−/−^ mice (Figure 4A). Loss of RESIST also restored the IFNγ response, based on CXCL9 and NOS2 staining (Figures 4B, S5A), and consequently fully rescued *Sp140*^−/−^ susceptibility in terms of CFU burden (Figure 4C) and survival (Figure 4D). This rescue occurred without impairing ‘normal’ IFNAR signaling, indicating that a normal (transient, modest) type I IFN response does not impair IFNγ responsiveness or *Mtb* control. Instead, strong and/or sustained type I IFN responses specifically mediate susceptibility to *Mtb*.

## DISCUSSION

Type I IFNs consistently correlate with human TB disease progression^16–20^ and cause susceptibility to *Mtb* in numerous mouse models^21–30^. Type I IFNs have been shown to impair the protective IL-1 response via multiple mechanisms^29,31,63^, but also promote TB susceptibility via IL-1-independent mechanisms^32,33,63^. Here, we provide evidence that sustained and strong (but not basal) type I IFN signaling cell intrinsically impairs IFNγ responsiveness in *Mtb*-infected lung macrophages. We further show that this impairment is an early consequence of type I IFN signaling that renders IMs permissive for intracellular *Mtb* replication, initiating the loss of bacterial control and disease susceptibility.

Previous *in vitro* studies have shown that type I IFNs can impair induction of selected IFNγ-responsive genes^44–47^. However, many ISGs are induced by both type I IFNs and IFNγ, complicating efforts to determine whether type I IFNs broadly suppress IFNγ responses. We show that IFNβ pre-exposure suppresses over 90% of genes induced at least two-fold more strongly by IFNγ than IFNβ. We also find that impairment of IFNγ signaling persists well after active type I IFN signaling subsides, requires the IFNAR-dependent transducers STAT2 and IRF9, and is conserved in mouse and human primary macrophages.

In human *Mycobacterium leprae* infections, elevated type I IFN signatures correlate with impaired IFNγ responses and poor clinical outcomes^48^. For *Mtb*, we recently reported that impaired IFNγ responses correlate with elevated type I IFNs in *Sp140*^−/−^ mice^27^, but a causal relationship had not been established. Based on our *in vitro* stimulation experiments and genetic evidence in mice, we identified CXCL9 as a robust marker of IFNγ responsiveness in IMs during *Mtb* infection (Figure 1), consistent with its use in another recent study^64^. By comparing CXCL9 induction in *Mtb*-infected B6, *Sp140*^−/−^, *Sp140^−/−^Ifnar1*^−/−^ and bone marrow chimera mice at day 25-28 post infection, we found that type I IFN signaling causally and cell-intrinsically impairs IFNγ responsiveness *in vivo* (Figures 1, 2). However, at this timepoint (also used in prior studies^27^), bacterial burdens differ between genotypes, confounding our ability to distinguish a direct inhibitory effect of IFNAR signaling on IFNγ responses from indirect effects of increased bacterial loads. To resolve this, we performed a time-course analysis (Figure 3), revealing a clear temporal progression in which early type I IFN signaling precedes and suppresses the later-emerging IFNγ response, ultimately elevating bacterial burdens. We also analyzed mixed chimeric mice, in which IFNAR-sufficient and -deficient cells respond within the same inflammatory environment. These experiments showed that IFNAR signaling cell-intrinsically represses IFNγ responsiveness and drives *Mtb* susceptibility independent of bacterial burdens.

The mechanism by which type I IFNs impair IFNγ signaling has been studied extensively, but no single dominant mechanism has emerged. Type I IFNs have been proposed to suppress IFNγ receptor expression^47,49^. Consistent with this, our RNA-seq analysis revealed a two-fold reduction in *Ifngr1* expression following IFNβ stimulation, and we previously reported reduced IFNγ receptor expression in SP140-deficient mice^27^. However, whether reduced receptor expression alone accounts for the observed impairment remains unclear, and additional, potentially redundant mechanisms likely contribute. For example, the shared signal transducer STAT1, acting downstream of both IFNAR and IFNGR, may become limiting under sustained type I IFN signaling^65^. IFNβ also induces SOCS1, a well-established negative regulator of IFNγ signaling. Beyond these, type I IFNs induce a broad antiviral response including ISGs that suppress gene expression, such as PKR-mediated eIF2α phosphorylation, IFIT-dependent inhibition of translation initiation, and OAS–RNase L–mediated RNA degradation^66^. Thus, type I IFN-mediated suppression of IFNγ signaling is likely multifactorial, arising from receptor-level regulation, shared signaling constraints, negative feedback, and global repression of gene expression.

A major unresolved question has been why type I IFNs promote TB pathogenesis in certain mouse strains (e.g., C3HeB/FeJ^29,67^ or 129^68^) but not in B6 mice. This has been puzzling because B6 mice are capable of mounting a robust type I IFN response^69^ and are generally highly resistant to viral infections^70–72^. Type I IFNs are expressed at higher levels in susceptible mouse strains during *Mtb* infection, but because *Mtb* itself induces type I IFNs^39,73^, it has been difficult to determine whether mice are susceptible due to higher interferon levels, or whether higher bacterial burdens in susceptible mice instead cause the increased interferon responses. Deleting *Ifnar* in susceptible mice^29,68^ restores resistance, but these experiments cannot address whether elevated IFN levels drive susceptibility, because *Ifnar* deletion eliminates all type I IFN signaling rather than selectively removing elevated levels while preserving basal responses. To gain insight into the quantitative dynamics by which type I IFN signaling impairs IFNγ responses and promotes *Mtb* susceptibility, we crossed *Sp140^−/−^* mice to mice harboring the sensitive *Mx1*-GFP reporter, in which type I IFN signaling induces GFP expression^59^. With these mice, we show that IFNγ responsiveness is not determined simply by the presence or absence of type I IFN signaling, but instead depends critically on the magnitude and/or duration of the response (Figure 3): strong and/or sustained type I IFN signaling specifically suppresses IFNγ signaling and promotes *Mtb* susceptibility. This was observed not only in *Sp140^−/−^* mice, but also, to a limited extent, in B6 mice, consistent with a recent report of *Mtb* strains inducing early detrimental type I IFN responses in B6 mice^60^. Our time course analysis shows that the enhanced type I IFN response in *Sp140^−/−^* versus wild-type mice precedes the divergence in bacterial burdens, and is thus not a consequence of elevated bacterial burdens but instead an important driver of susceptibility to *Mtb*. This conclusion is supported not only by the Mx1-GFP reporter data, but also by independent genetic experiments (Figure 4) in which we deleted the *Resist1/2* locus, which we previously showed encodes a positive regulator of type I IFN production specifically de-repressed in *Sp140^−/−^* mice^35^. *Resist1/2* deletion had no detectable effect in the B6 background (where it is not normally expressed) and did not affect basal type I IFN levels. However, in *Sp140^−/−^* mice, RESIST deficiency restored an appropriately transient type I IFN response, permitting IFNγ-dependent *Mtb* restriction comparable to that in B6 mice.

Our findings emphasize that IFNγ production does not necessarily translate into effective IFNγ signaling. Instead, IFNγ responsiveness is shaped by the surrounding cytokine environment, particularly the presence of type I IFNs. Accordingly, assessing IFNγ activity requires measuring downstream responses rather than IFNγ levels alone. Here, we provide robust markers to reliably quantify IFNγ responsiveness and a clear example, in the context of TB, of why this distinction is critical. Together, our findings may help explain why induction of IFNγ or IFNγ-producing CD4^+^ T cells^11–15^ is not always sufficient to confer protection against *Mtb* or other pathogens where type I IFN-driven susceptibility occurs, and point to strategies for enhancing IFNγ-mediated immunity and host-directed therapies.

## RESOURCE AVAILABILITY

### Lead Contact

Further information and requests for resources and reagents should be directed to and will be fulfilled by the lead contact, Russell E. Vance (rvance@berkeley.edu).

### Materials Availability

Materials used in this study will be provided upon request and available upon publication.

## STAR METHODS

### EXPERIMENTAL MODEL

#### Mice

Mice were maintained in accordance with the regulatory standards of the University of California Berkeley Institutional Animal Care and Use Committee under specific pathogen-free conditions. In all experiments mice were age- and sex-matched and were 8-18 weeks old at the start of the infections. Every experiment included female and male mice. Mouse lines used are C57BL/6J (B6), B6.129S2-Ifnar1^tm1Agt^/Mmjax (*Ifnar1^−/−^*), B6.129S7-Ifngr1tm1Agt/J (*Ifngr1^−/−^*), C57BL/6J-Ptprcem6Lutzy/J (CD45.1), B6.Cg-Mx1tm1.1Agsa/J (*Mx1^gfp^*), B6(Cg)-Ifnar1tm1.1Ees/J (*Ifnar1^fl/fl^*) and C57BL/6N-Ifngr1tm1.1Rds/J (*Ifngr1^fl/fl^*) that were purchased from Jackson Laboratories. *Sp140^−/−^* previously made^34^ was crossed in house to *Ifnar1^−/−^*, CD45.1 and *Mx1^gfp^* to generate *Sp140^−/−^ Ifnar1^−/−^*, *Sp140^−/−^*CD45.1 and *Sp140^−/−^Mx1^gfp^*, respectively. B6-Fcgr1tm2Ciphe (CD64^Cre^) was previously described^74^. *Ifnar1^fl/fl^* and *Ifngr1^fl/fl^* were crossed to *Sp140^−/−^* and CD64^Cre^ to generate *Sp140^−/−^Ifnar1^fl/fl^*CD64^Cre^ and *Ifngr1^fl/f^* CD64^Cre^. *Sp140^−/−^Resist^−/−^* were previously described^35^ and backcrossed in house to B6 to generate *Resist^−/−^*. *Sp140^−/−^Irf9^−/−^* and *Sp140^−/−^Stat2^−/−^* mice were generated in this study as described below.

#### Mouse bone marrow macrophages (BMMs)

Bones (femurs and tibias) from B6 or *Ifnar1^−/−^* mice were harvested and sterilized in 70% EtOH. Bone marrow was isolated by flushing the bones with ice-cold BMM media consisting of DMEM supplemented with 10% fetal bovine serum (FBS), 10% MCSF (generated from 3T3 cells), GlutMax, 10mM HEPES and Pen-Strep (Thermo). Isolated bone marrow was filtered through a 70μm cell strainer, centrifuged at 4°C for 5 min, 600G, resuspended in BMM media and seeded into 6-8 15cm non-treated petri dishes. Cells were incubated for 7 days to differentiate into BMMs with adding 50% BMM media on day 3 before transferring cells into the appropriate multi-well format using a cell scraper. After 2 days of resting, BMMs were exposed to cytokines according to the experimental setup.

#### Human macrophages

THP-1 cells (ATCC) were maintained in RPMI including 10% FBS, GlutMax and Pen-Strep (complete RPMI). THP-1 were differentiated by adding 100 ng/mL phorbol myristate acetate (PMA, Invivogen, tlrl-pma) for 48h followed by 36h rest before used for exposure assays. Cryopreserved negatively selected primary human monocytes were purchased from AllCells and differentiated for 6 days in complete RPMI supplemented with 50 ng/ml human M-CSF (PeproTech, 300-25). Cells were lifted with trypsin and transferred into a 96-well format for exposures.

### METHOD DETAILS

#### Generation of *Sp140^−/−^Irf9^−/−^* and *Sp140^−/−^Stat2^−/−^* mice

*Sp140^−/−^Irf9^−/−^* and *Sp140^−/−^Stat2^−/−^* mice were generated by electroporation of *Sp140*^−/−^ zygotes with Cas9 and sgRNA UACGCUGCACCCGAAAGCUG and AGUGGUCCCACUGGUUCAGU, respectively. Founders were genotyped and backcrossed to *Sp140^−/−^* mice, and progeny with matching alleles were further bred. Genotyping was performed by sequencing of PCR product with the primer pairs CAGGGGTTTGCAAGTTGTTG, AGACATGGTTGGTTCTACTTTCT for *Irf9* and GGCTCATCTGATTTCAGGCC, CCTCTCAGGTGACACACAAC for *Stat2*. Established knock-out mouse lines had a 20, 17 base pair deletion in *Irf9* exon 3, *Stat2* exon 3, respectively.

#### Mouse *Mtb* infections, *in vivo* antibody mediated blockage, tissue processing for CFU and flow cytometry analysis

*Mtb* Erman strain including the ones constitutively expressing either mWasabi or mCherry have previously been described^27^. Inoculum was prepared from a frozen stock and diluted in 9ml sterile PBS at an of ca. OD of 0.002. Mice were inserted into an aerosolizer device (Glas-Col, Terre Haute, IN) and infected through the aerosol route at a low dose of ca. 20-100 CFUs. Infectious dose was verified from 2-3 mice at day 1 post infection. For IFNAR1 blockage *in vivo* 500ug anti-IFNAR1 antibody (bioXcell, MAR1-5A3) per mouse was injected intraperitoneal every other day starting at day 7 pi. Mice were sacrificed at indicated time points post infections and the complete lungs were harvested into a GentleMACS C tube (Miltenyi Biotec) with 2ml digestion media consisting of 2ml RPMI media (Gibco) with 30ug/ml DNase I (Roche), 70ug/ml Liberase TM (Roche) and Brefeldin A (BioLegend). Lungs were cut into large pieces using the program lung_01 on GentleMACS device (Miltenyi Biotec) before incubating at 37°C for 30 minutes. Lungs were homogenized with program Lung_2 on the GentleMacs device and digestions was stopped by adding 2ml of PBS including 20% Newborn Calf Serum (Thermo Fisher Scientific). Lung homogenate was filter through a 70μm SmartStrainers (Miltenyi Biotec) into 15ml falcon tube. From this single cell suspension 100ul was saved for CFU plating assay, whereas the rest centrifuged at 1,600 rpm for 8 minutes at 4°C. Cell pellet was resuspended in FACS buffer and used for flow cytometry analysis.

#### CFU plating

7H11 plates supplemented with 10% BD BBL^™^ Middlebrook OADC Enrichment (Fisher) and 0.5% glycerol were prepared ahead of time and stored at 4°C. Lung homogenates were serial diluted in PBS and 50ul of appropriate dilutions were plated. After 3 weeks incubation at 37°C, CFUs were enumerated to back calculate CFUs per lung.

#### Flow cytometry of lung homogenate

The following fluorophore-coupled antibodies were used accordingly together with fixable viability dye (Ghost Dye^™^ Violet 780; Tonbo Biosciences), TruStain FcX PLUS (S17011E, BioLegend), Super Bright Complete Staining Buffer (Thermo Fisher Scientific) and True-Stain Monocyte Blocker (BioLegend) to prepare a Master Mix and stain the single cells suspension from above: BV421-coupled MHCII (M5/114.15.2, BioLegend), BV480-coupled B220 (RA3-6B2, BD Biosciences), BV480-coupled CD90.2 (53-2.1, BD Biosciences), BV605-coupled CD64 (X54-5/7.1, BioLegend), BV711-coupled CD11b (M1/70, BioLegend), BV785-coupled Ly6C (HK1.4, BioLegend), PE-Cy7-coupled MerTK (DS5MMER, Thermo Fisher Scientific), APC-R700-coupled Siglec F (E50-2440, BD Biosciences), BUV496-coupled CD45 (30-F11, BD Biosciences), BUV563-coupled Ly6G (1A8, BD Biosciences), BUV737-coupled CD11c (HL3, BD Biosciences), BV421-coupled CD45.1 (A20, BioLegend), BUV496-coupled CD45.2 (104, BD Biosciences). Staining was performed at room temperature for >30 minutes before washing the cells three times with FACS buffer. Stained cells were fixed with Cytofix/Cytoperm (BD Biosciences) for >30 minutes at room temperature before retrieved from BSL3 facility. Fixed cells were permeabilized by washing cells four times with Permeabilization Buffer (Invitrogen) before performing intracellular staining using PE-coupled Viperin (MaP.VIP, BD Biosciences), AF647-coupled CXCL9 (MIG-2F5.5, BioLegend) and BUV395-coupled NOS2 (CXNFT, Invitrogen). Cells were run on an Aurora (Cytek) flow cytometer and analyzed with Flowjo version 10 (BD Biosciences).

#### Confocal Microscopy for imaging and image analysis

For microscopy the middle lobe was harvested and directly fixed with Cytofix/Cytoperm (BD Biosciences) diluted in PBS (1:2) for >24h at 4°C before retrieving from BSL3 laboratory. Fixed lung tissues were washed with PBS and dehydrated for >12h at 4°C in PBS containing 20% sucrose. Lung tissues were embedded in O.C.T. (Tissue-Tek) and stored at −80°C. 10μm sections were prepared using a Leica CM3050S Cryotome and mounted on Superfrost Plus Microscope Slides (Fisher). Sections were rehydrated with PBS, permeabilized with PBS containing 0.5% Tx-100 and blocked with 10% Normal Goat Serum (Vector Laboratories) before staining with DAPI (Sigma) and fluorophore-coupled antibodies. Stained tissue was covered with a cover slip and VectaShield HardSet (Vector Laboratories). A Zeiss LSM710 confocal microscope and FIJI software was used for image analysis. Shortest distance analysis was performed with a FIJI plugin for distance analysis called DiAna^75^.

#### Confocal microscopy and image processing for histo-cytometry analysis

Confocal Microscopy was performed using a Zeiss LSM 880 laser scanning confocal microscope (Zeiss) equipped with two photomultiplier detectors, a 34-channel GaASP spectral detector system, and a 2-channel AiryScan detector as well as 405, 458, 488, 514, 561, 594, and 633 lasers. Stained 20μm paraformaldehyde fixed lung sections from *Mtb*-mCherry infected mice were inspected with a 5× air objective to find representative lesions and distal sites and then imaged using a 63× oil immersion objective lens with a numerical aperture of 1.4. For each infected lung, one 12-15 x 12-15 tiled Mtb-heavy lesion image and one 4×4 tiled distal site image was taken consisting of 20μm z-stacks with a 1.5μm step size. Additionally, the Zeiss LSM 880 microscope was used to image single color-stained Ultracomp eBeads Plus (Thermo Fisher Scientific) for generating a compensation matrix. Image analysis was performed using Chrysalis software.^76^ Briefly, a compensation matrix was generated by automatic image-based spectral measurements on single color-stained controls in ImageJ by using Generate Compensation Matrix script. This compensation matrix was used to perform linear unmixing on three-dimensional images with Chrysalis. Chrysalis was also used for further image processing, including rescaling data and generating new channels by performing mathematical operations using existing channels. For histo-cytometry analysis, Imaris 9.9.1 (Bitplane) was used for surface creation to digitally identify cells in images based on protein expression.^77^ Total number, surface volume, and mean fluorescence intensity of each cell type within a given surface were calculated, along with total volume of each z-stacked section. Statistics for identified cells were exported from Imaris and then imported into FlowJo version 10 (BD Biosciences) for quantitative image analysis.

#### Bone marrow chimeras

From donor mice, bones (femurs and tibias) were harvested, sterilized in 70% EtOH and bone marrow was isolated by flushing the bones with cold PBS using a syringe and filtering through a 70μm cell strainer. Cells were once washed and resuspended in PBS for injection. For mixed bone marrow chimeras, bone marrow cells from different genotypes were mixed in a 1:1 ratio. Recipient mice were lethally irradiated two times within 12-20 hours with a Precision X-Rad320 X833 Ray irradiator (North Branford, CT). Mice received 2-5 Mio bone marrow cells in 200ul PBS by retro-orbital injection. Mice were housed for >8 weeks to allow hematopoietic reconstitution prior *Mtb* infection.

#### Cytokine exposures of cells for flow cytometry analysis

For exposures, cytokines expressed in either HEK293 or CHO cells were used. Specifically, if not other stated in figures or figure legends, 2ng/ml mouse IFNβ (581304, BioLegend), 50ng/ml mouse IFNγ (ab259378, Abcam), 10ng/ml mouse TNF (ab259411, Abcam), 2ng/ml human IFNβ (300-02BC, Thermo), 50ng/ml human IFNγ (ab259377, Abcam). TLR agonist used were 50ng/ml Pam3CSK4 (Invivogen, tlrl-pms) and 10ng/ml LPS (Invivogen, tlrl-3pelps).

#### Flow cytometry of cytokine exposed macrophages

Brefeldin A was added to the BMM media for the last 4 hour of cytokine exposure. BMM media was removed, and BMMs were incubated for 20min with pre-warmed PBS with 4mM EDTA. Detached BMMs were transferred and washed once in PBS prior staining with fixable viability dye (Ghost Dye^™^ Violet 780; Tonbo Biosciences). After 30min incubation at room temperature, cells were washed three times with PBS and fixed with IC Fixation Buffer (eBioscience) for 15 minutes at room temperature. Cells were washed and fixed four times with Permeabilization Buffer (Invitrogen) before performing intracellular staining using PE-coupled Viperin (MaP.VIP, BD Biosciences), AF647-coupled CXCL9 (MIG-2F5.5, BioLegend) and AF488-coupled NOS2 (CXNFT, eBiosciences). Cells were run on a Fortessa (BD Biosciences) flow cytometer and analyzed with Flowjo version 10 (BD Biosciences). All the analysis were performed on viable cells only.

#### Cas9-ribonucleoprotein (RNP) mediated gene disruption in BMMs

Gene disruption in BMMs was performed with Cas9-ribonucleoprotein (RNP) electroporation at day 5 post seeding as described previously^78^. In brief, Cas9 2 NLS nuclease (Synthego) was pre-incubated with gRNAs (Synthego, sgRNA EZ kits) and Alt-R Cas9 Electroporation Enhancer (IDT, 1075916) for >20min at room temperature to form Cas9-RNPs. BMMs were lifted using a cell scraper, washed in PBS and resuspended in Lonza P3 buffer (Lonza, V4XP-3032) including Supplement 1 according to manufacturer’s protocol before combining with Cas9-RNPs. Cells were electroporated with the Lonza 4D-Nucleofector Core Unit (AAF-1002B) using the program CM-137. Electroporated BMMs were recovered in BMM media and 2Mio cells were plated per 10cm non-treated petri dish and incubated at 37°. 50% fresh media was added two days post electroporation and after 4 days of recovery edited BMMs were transferred into the appropriate multi-well format for exposure assays. The following gRNA sequences were used: Ifnar2: CAGACGGUGUGAUAGUCUCU & CAAAGACGAAAAUCUGACGA, Stat2: AGUGGUCCCACUGGUUCAGU, Irf9: UACGCUGCACCCGAAAGCUG & GUUGUAAACCACUCAGACAG.

#### Bulk RNA-seq sample preparation and analysis

For exposures 10ng/ml mouse IFNβ (581304, BioLegend), 10ng/ml mouse IFNγ (ab259378, Abcam) was used. Upon cytokine exposure, BMMs were lysed with TRK lysis buffer (Omega Bio-Tek) including 2-mercaptoethanol (Thermo Fisher Scientific). Total RNA isolation was performed using the E.Z.N.A Total RNA Kit I (Omega Bio-Tek) with a DNase treatment on-column (Qiagen, 79254). The library preparation, sequencing, and read alignment to the mouse genome was performed by Azenta Life Sciences. Raw counts were used as input for analysis with DESeq2^79^.

#### RT-qPCR analysis

Total RNA was isolated the same way as for bulk RNAseq described above. cDNA was reverse transcribed from RNA with Superscript III Reverse Transcriptase (Invitrogen, 18080093) and oligo dT18 (NEB, S1316S) in the presence of RNase inhibitors. Diluted cDNA was assessed by RT-qPCR using the Power SYBR Green PCR Master Mix (Thermo Fisher Scientific, 43-676-59) in technical duplicates. PrimeTime qPCR Primers (IDT) were used: Cxcl9 Mm.PT.58.5726745, Rsad2: Mm.PT.58.11280480, Actin: Mm.PT.39a.22214843.g.

### QUANTIFICATION AND STATISTICAL ANALYSIS

Statistical tests to determine statistical significance were performed using Prism (GraphPad) software and are indicated in the figure legends. *p < 0.05, **p < 0.01, ***p < 0.001, ns = not significant.

## Supplementary Material

1 Figure S1. Analysis of type I IFN-driven suppression of IFNγ signaling, related to Figure 1
(**A**) Log_2_ fold change of IFNγ signature genes in bone marrow derived macrophages (BMMs) upon exposure to IFNγ ± IFNβ pre-exposure (*n* = 3). (**B**) Induction of IFN-specific genes by BMMs upon IFNβ and IFNγ. Genes two-fold more induced upon IFNγ compared to IFNβ are defined as IFNγ specific genes (blue). Average of *n* = 3 is plotted in (B). (**C**) Table of the IFNγ specific genes that are not inhibited by type I IFN signaling. (**D**) Quantification of CXCL9 mRNA levels by RT-qPCR from BMMs exposed to IFNγ ± IFNβ-preexposure. UT = Untreated (**E**) Representative flow cytometry histogram of CXCL9 from WT and *Ifnar1^−/−^* BMMs exposed to IFNγ ± IFNβ-preexposure. (**F**) Quantification of viable BMMs upon exposure to cytokines. (**G**) Quantification of CXCL9 protein expressing BMMs from WT mice exposed to 10 and 100ng/ml IFNγ ± IFNβ-preexposure. (**H**) Quantification of CXCL9 protein expressing BMMs from WT mice upon exposure to IFNβ, IFNγ or IFNβ/γ in the absence or presence of tumor necrosis factor (TNF) and Toll-like receptor agonists Pam3CSK4 (PAM) or Lipopolysaccharide (LPS). (**I**) Quantification of *Rsad2* mRNA (encoding Viperin) levels by RT-qPCR of WT BMMs directly upon IFNβ exposure or after removing it for 8h. (**J**) Quantification of CXCL9 protein expressing BMMs from WT mice exposed to IFNγ at various time post transient overnight IFNβ-exposure. (**K**) Quantification of NOS2 protein expressing BMMs from WT mice upon exposure to IFNβ, IFNγ or IFNβ/γ in the absence or presence of TNF and Toll-like receptor agonists PAM or LPS. (**L**) Quantification of CXCL9 protein expressing human THP-1 macrophages exposed to IFNγ ± IFNβ-preexposure. (D, F-L) Representative data from ≥ 3 biological replica with (D, F, H-L) n ≥ 2 and with (G) n = 1 per condition. Statistical significance was calculated in (A) with RM two-way ANOVA with the Geisser-Greenhouse correction and Sidak’s multiple comparison test. *p < 0.05, **p < 0.01, ***p < 0.001, ns = not significant.
Figure S2. Control data and NOS2 expression in IMs of *Mtb* infected mice, related to Figure 1
(**A**) Representative flow cytometry plots of a *Mtb*-mWasabi infected B6 mouse gated on live CD45^+^ B220^−^ CD90.2^−^ cells to identify interstitial macrophages (CD64^+^ MerTK high SiglecF^−^), which can be grouped into bystander (mWasabi^−^) and infected (mWasabi^+^) cells. (**B**) Representative flow cytometry plots of CXCL9 expression by IMs and neutrophils at day 28-29 pi and in (**C**) corresponding quantification. (**D**) Representative flow cytometry plots of CXCL9 vs autofluorescence (AF) in indicated mouse strains at day 25-26 pi. (**E**) Quantification of CXCL9 MFI of IMs in fully stained and fully stained minus CXCL9 (FMO CXCL9) samples from *Mtb*-infected B6 and *Sp140^−/−^* mice at day 25 pi. (**F**) MFI of CXCL9 and (**G**) MFI of NOS2 in IMs from *Mtb*-infected B6 and *Ifngr1^−/−^* mice at day 25 pi grouped in bystander and infected IMs. (**H**) MFI of NOS2 staining in IMs from *Mtb*-infected B6 and *Sp140^−/−^* mice at day 25 pi. (**I**) MFI of NOS2 staining in IMs from *Mtb*-infected *Sp140^−/−^*, *Sp140^−/−^Ifnar1^−/−^*, *Sp140^−/−^Irf9^−/−^* and *Sp140^−/−^Stat2^−/−^* mice at day 28-29 pi. (**J**) MFI of NOS2 staining in IMs from *Mtb*-infected B6 and B6.*Ifnar1^−/−^* mice at day 25-26 pi. (C, E, F-J) Data was combined from ≥ 2 biological replica with n ≥ 6 per group. Statistical significance was calculated in (E-G) with Two-way ANOVA and Sidak’s multiple comparison test, in (I) with Brown-Forsythe and Welch ANOVA test and Dunnett’s T3 multiple comparisons test, in (C, H, J) with Welch’s t-test. *p < 0.05, **p < 0.01, ***p < 0.001, ns = not significant.
Figure S3. Control data, related to Figure 2
(**A**) Representative flow cytometry plots of Viperin vs autofluorescence (AF) in indicated mouse strains at day 25-26pi. (**B**) Quantification of Viperin MFI of IMs in fully stained and fully stained minus Viperin (FMO Viperin) samples from *Mtb*-infected B6 and *Sp140^−/−^* mice at day 25 pi. (**C-F**) Analysis of *Mtb*-infected mixed *Sp140^−/−^* bone marrow chimeras containing CD45.1- and CD45.2-positive cells, either both IFNAR-proficient (control) or IFNAR-proficient and IFNAR-deficient, respectively, at day 25-26 pi. Comparison in terms of (**C**) Viperin positivity, (**D**) CXCL9 MFI, (**E**) percent infected IMs and (**F**) *Mtb*-mWasabi MFI. (**G**) CXCL9 MFI analysis of Viperin-negative and -positive IMs from *Sp140^−/−^Ifnar1^+/+^* subpopulation in figure 3J. Data in (B) from one biological replica with n = 5 per group and in (C-G) from ≥ 2 biological replica with n ≥ 6 per group. Statistical significance was calculated in (B) with Two-way ANOVA and Sidak’s multiple comparison test, in (C-G) with paired t-test. *p < 0.05, **p < 0.01, ***p < 0.001, ns = not significant.
Figure S4. Control data for *Mx1^gfp^* mouse line and T cell recruitment in B6 vs *Sp140^−/−^* mice, related to Figure 3
(**A**) Quantification of Mx1-expression of BMMs established from type I IFN-signaling reporter mouse line (*Mx1^gfp^*) upon exposure to IFNβ compared to background fluorescence. (**B**) MFI of Mx1-GFP expression in IMs from *Mtb*-infected *Mx1^gfp^* mice without or with administration of anti-IFNAR1 antibodies. (**C**) Quantification of *Mtb*-mCherry-positive cells from *Mtb*-infected *Sp140^+/+^ or Sp140^+/−^* and *Sp140^−/−^* mice without or with administration of anti-IFNAR1 antibodies at day 18 pi. (**D**) Representative micrograph with CD4^+^ T cells of infected lung lesions from *Mtb*-infected B6, *Sp140^−/−^* and *Sp140^−/−^* treated with anti-IFNAR1 antibodies at day 18 pi. (**E**) Shortes distance quantification of CD4^+^ T cells to *Mtb* within infected lesions at day 18 pi. (**F**) Representative micrograph with T cells of lung lesions from *Mtb*-infected B6, *Sp140^−/−^* and *Sp140^−/−^* treated with anti-IFNAR1 antibodies at day 28 pi. (**G**) Quantification of CD4^+^ T cells per 10^6^ μm^3^ in “Infected Lesions” and “Healthy Tissue” from B6 and *Sp140^−/−^ Mtb*-infected mouse lungs at day 25 pi. Data in (A) from one biological replica with n = 2 per group and in (B, C, E, G) from ≥ 2 biological replica with n ≥ 3 per group. Statistical significance was calculated in (B) with Brown-Forsythe/Welch ANOVA test and Dunnett’s T3 multiple comparisons test, in (C) with Kruskal-Wallis test and Dunn’s multiple comparison test, in (G) with an ordinary two-way ANOVA and Turkey’s multiple comparisons test. *p < 0.05, **p < 0.01, ***p < 0.001, ns = not significant.
Figure S5. NOS2 expression in *Resist*-deficient mice, related to Figure 4
(**A**) MFI of NOS2 staining in IMs from *Mtb*-infected B6, *Resist^−/−^*, *Sp140^−/−^*, *Sp140^−/−^ Resist^−/−^* mice at day 26-28 pi. Statistical significance was calculated with Brown-Forsythe/Welch ANOVA test and Dunnett’s T3 multiple comparisons test. *p < 0.05, **p < 0.01, ***p < 0.001, ns = not significant.

## Figures and Tables

**Table:** Key resources table

REAGENT or RESOURCE	SOURCE	IDENTIFIER
Antibodies		
Anti-mouse IFNAR-1 clone MAR1-5A3	BioXCell	Cat #BE0241; RRID: AB_2687723
TruStain FcX PLUS (anti-mouse CD16/32 ) clone S17011E	BioLegend	Cat # 156604; RRID:AB_2783138
BV421 anti-mouse MHCII clone M5/114.15.2	BioLegend	Cat # 107631; RRID:AB_10900075
BV480 anti-mouse B220 clone RA3-6B2	BD Biosciences	Cat # 565631; RRID:AB_2739311
BV480 anti-mouse CD90.2 clone 53-2.1	BD Biosciences	Cat # 566082; RRID:AB_2739494
BV605 anti-mouse CD64 clone X54-5/7.1	BioLegend	Cat # 139323 PRID:AB_2629778
BV711 anti-mouse CD11b clone M1/70	BioLegend	Cat # 101241 PRID:AB_11218791
BV785 anti-mouse Ly6C clone HK1.4	BioLegend	Cat # 128041; RRID: AB_2565852
PE-Cy7 anti-mouse MerTK clone DS5MMER	Thermo Fisher Scientific	Cat # 25-5751-82; RRID:AB_2573466
APC-R700 anti-mouse Siglec F clone E50-2440	BD Biosciences	Cat # 565183; RRID:AB_2739097
BUV496 anti-mouse CD45 clone 30-F11	BD Biosciences	Cat # 749889; RRID:AB_2874129
BUV563 anti-mouse Ly6G clone 1A8	BD Biosciences	Cat # 612921; RRID:AB_2870206
BUV737 anti-mouse CD11c clone HL3	BD Biosciences	Cat # 612796; RRID:AB_2870123
BV421 anti-mouse CD45.1 clone A20	BioLegend	Cat # 110731 PRID:AB_10896425
BUV496 anti-mouse CD45.2 clone 104	BD Biosciences	Cat # 741092 RRID:AB_2870691
PE anti-human/mouse Viperin clone MaP.VIP	BD Biosciences	Cat # 565196; PRID:AB_2739106
AF647 anti-mouse CXCL9 clone MIG-2F5.5	BioLegend	Cat # 515606; PRID:AB_1877135
BUV395 anti-mouse NOS2 clone CXNFT	Invitrogen	Cat # 363-5920-82; PRID:AB_2942115
APC anti-human CXCL9 clone J1015E10	BioLegend	Cat # 357906; PRID:AB_2566034
Bacterial and virus strains		
Bacteria: Mtb strain Erdman	A gift from Sarah Stanley, University of California Berkeley	N/A
Bacteria: Mtb-Wasabi	Kotov et al.^27^	N/A
Bacteria: Mtb-mCherry	Kotov et al.^27^	N/A
Biological samples		
Human monocytes	AllCells	Donor ID: 889009867
Chemicals, peptides, and recombinant proteins		
DNase I	Roche	Cat # 11284932001
Liberase TM	Roche	Cat # 5401127001
Brefeldin A	BioLegend	Cat # 420601
Phorbol myristate acetate (PMA)	Invivogen	Cat # tlrl-pma
Human M-CSF	PreproTech	Cat # 300-25
Middlebrook OADC	Thermo Fisher Scientific	Cat # b12351
Hygromycin B Gold	Invivogen	Cat # ant-hg-1
Kanamycin	Millipore Sigma	Cat # K4000-5G
M-CSF	Vance lab	N/A
TRK lysis buffer	Omega Bio-Tek	Cat # PR021
2-mercaptoethanol	Thermo Fisher Scientific	Cat # 21985023
Ultracomp eBeads Plus	Thermo Fisher Scientific	Cat # 01-3333-42
Super Bright Complete Staining Buffer	Thermo Fisher Scientific	Cat # SB-4401-75
True-Stain Monocyte Blocker	BioLegend	Cat # 426102
Cytofix/Cytoperm	BD biosciences	Cat # 554722
AccuCheck Counting Beads	Invitrogen	Cat # PCB100
Middlebrook 7H9 Broth (Dehydrated)	Thermo Fisher Scientific	Cat # R454012
BBL seven H11 agar base	BD biosciences	Cat # 212203
SpCas9 2 NLS nuclease	Synthego	N/A
Alt-R^®^ Cas9 Electroporation Enhancer	IDT	Cat # 1075916
Ghost Dye Violet 780	Tonbo Boesciences	Cat # SKU 13-0865-T100
eBioscience Permeabilization Buffer (10X)	Invitrogen	Cat # 00-8333-56
Tissue-Tek O.C.T. Compound	VWR International	Cat # 25608-930
Normal Goat Serum Blocking Solution	Vector Laboratories	Cat # S-1000-20
VECTASHIELD^®^ HardSet^™^ Antifade Mounting Medium	Vector Laboratories	Cat # H-1400-10
Mouse IFNβ	BioLegend	Cat # 581304
Mouse IFNγ	Abcam	Cat # ab259378
Mouse TNF	Abcam	Cat # ab259411
Human IFNβ	Thermo Fisher Scientific	Cat # 300-02BC
Human IFNγ	Abcam	Cat # ab259377
Lipopolysaccharide (LPS)	Invivogen	Cat # tlrl-3pelps
Pam3CSK4	Invivogen	Cat # tlrl-pms
eBioscience IC Fixation Buffer	Thermo Fisher Scientific	Cat # 00-8222-49
Lonza P3 buffer	Lonza	Cat # V4XP-3032
DNase	Qiagen	Cat # 79254
Superscript III Reverse Transcriptase	Invitrogen	Cat # 18080093
oligo dT18	New England Biolabs	Cat # S1316S
Power SYBR Green PCR Master Mix	Thermo Fisher Scientific	Cat # 43-676-59
Critical commercial assays		
E.Z.N.A Total RNA Kit I	Omega Bio-Tek	Cat # R6834-02
Deposited data		
Bulk RNA-seq of cytokine stimulated mouse bone marrow-derived macrophages	Kotov et al.^27^	N/A
Experimental models: Cell lines		
THP-1 cells	ATCC	N/A
Experimental models: Organisms/strains		
Mouse: C57BL/6J	The Jackson Laboratory	Cat # 000664; RRID:IMSR_JAX:000 664
Mouse: *Ifnar1^−/−^*: B6.129S2-*Ifnar1^tm1Agt^/*Mmjax	The Jackson Laboratory	Cat # 007906 RRID:IMSR_JAX:007 906
Mouse: *Ifnar1^fl^*: B6(Cg)-*Ifnar1^tm1.1Ees^*/J	The Jackson Laboratory	Cat # 028256 RRID:IMSR_JAX:028 256
Mouse: CD64^Cre^: B6-*Fcgr1^tm2Ciphe^*	Scott et al.^80^	N/A
Mouse: *Sp140^−/−^*	Ji et al.^34^	N/A
Mouse: *Ifngr1^−/−^*: B6.129S7-*Ifngr1^tm1Agt^*/J	The Jackson Laboratory	Cat # 003288 RRID:IMSR_JAX:003 288
Mouse: CD45.1: C57BL/6J-*Ptprc^em6Lutzy^*/J	The Jackson Laboratory	Cat # 033076 RRID:IMSR_JAX:033076
Mouse: *Mx1^gfp^*: B6.Cg-*Mx1 ^tm1.1Agsa^*/J	The Jackson Laboratory	Cat # 033219 RRID:IMSR_JAX:033 219
Mouse: *Sp140^−/−^Resist^−/−^*	Witt et al.^35^	N/A
Mouse: *Sp140^−/−^Irf9^−/−^*	This paper	N/A
Mouse: *Sp140^−/−^Stat2^−/−^*	This paper	N/A
Mouse: *Ifngr1^fl^*: C57BL/6N-*Ifngr1^tm1.1Rds^*/J	The Jackson Laboratory	Cat # 025394 RRID:IMSR_JAX:025 394
Oligonucleotides		
sgRNA *Irf9* Nr 1: UACGCUGCACCCGAAAGCUG	This paper	N/A
sgRNA *Irf9* Nr 2: GUUGUAAACCACUCAGACAG	This paper	N/A
sgRNA *Stat2*: AGUGGUCCCACUGGUUCAGU	This paper	N/A
sgRNA *Ifnar2* Nr 1: CAGACGGUGUGAUAGUCUCU	This paper	N/A
sgRNA *Ifnar2* Nr 2: CAAAGACGAAAAUCUGACGA	This paper	N/A
PrimeTime qPCR Primers Cxcl9	Integrated DNA Technologies	Mm.PT.58.5726745
PrimeTime qPCR Primers Rsad2	Integrated DNA Technologies	Mm.PT.58.11280480
PrimeTime qPCR Primers Actin	Integrated DNA Technologies	Mm.PT.39a.2221484 3.g
Software and algorithms		
Chrysalis	Kotov et al.^76^	https://github.com/Histocytometry/Chrysalis
Generate Compensation Matrix	Kotov et al.^76^	https://github.com/Histocytometry/Chrysalis
Imaris version 9.9.1	Bitplane	N/A
FlowJo version 10	BD Biosciences	N/A
R version 3.16	R Development Core Team	http://www.r-project.org/
RStudio “Cherry Blossom” Release	Posit	https://posit.co/products/open-source/rstudio/
Adobe Illustrator	Adobe.com	N/A
Prism	GraphPad	N/A
ImageJ (FiJi)	https://imagej.net/Fiji	RRID:SCR_002285
BioRender	BioRender Company	https://BioRender.com/64uv53d https://BioRender.com/1dzqp93
Other		
GentleMACS	Miltenyi Biotec	N/A
Lonza 4D-Nucleofector Core Unit	Lonza	Cat # AAF-1002B
5 laser Aurora analyzer	Cytek	N/A
5 laser LSRFortessa analyzer	BD Biosciences	N/A
LSM 880 laser scanning confocal microscope	Zeiss	N/A
LSM 710 laser scanning confocal microscope	Zeiss	N/A

## Data Availability

Raw bulk RNA sequencing data are deposited in the NCBI Gene Expression Omnibus: GSE334520.

## Highlights

- Cell-intrinsic type I IFN signaling broadly impairs IFNγ responses
- Impaired IFNγ responsiveness compromises immunity to *Mtb*
- Type I IFNs render macrophages permissive to *Mtb* by suppressing IFNγ responses
- Only strong and sustained type I IFN signaling impairs *Mtb* immunity
