# Supplementary material for: Strong sustained type I IFN signaling acts cell intrinsically to impair IFNγ responses and cause tuberculosis susceptibility
Source: Cell Host Microbe. Author manuscript; Available in PMC 2026 Sep 1. (PMC13528601; doi:10.1016/j.chom.2026.07.014)
Supplement: Supplementary file 1 [file NIHMS2205886-supplement-1.pdf]

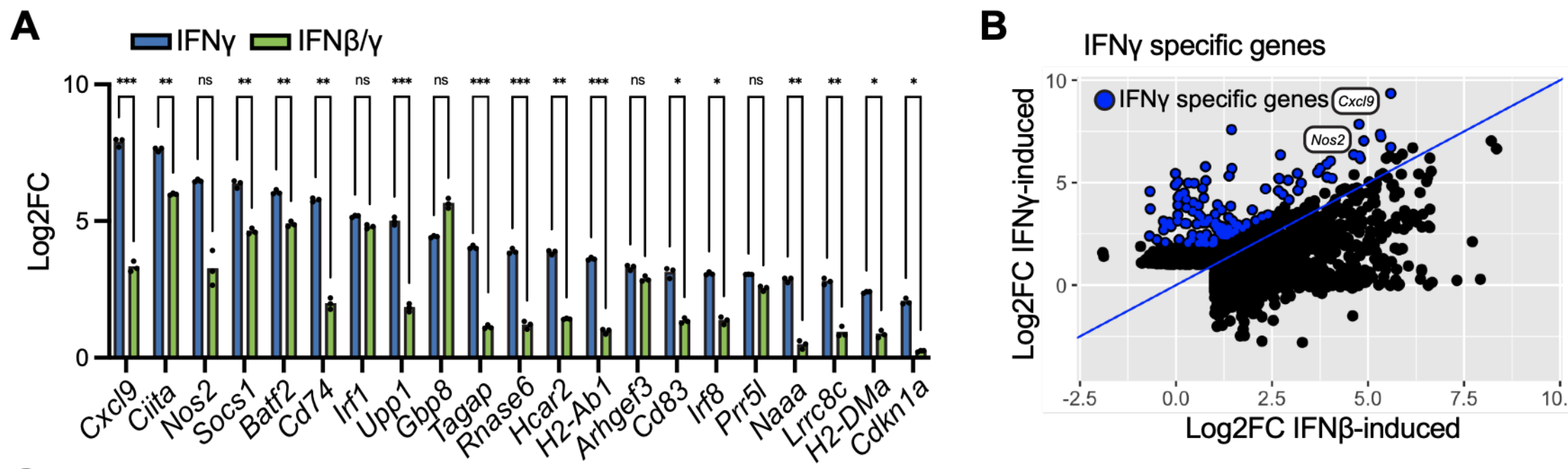

C

Gene	*Plbd1*	*Amotl2*	*Gbp10*	*Gbp8*	*Gbp2b*	*Gm40182*	*F830016B08Rik*	*Gm4841*	*Tnfsf13b*	*Mreg*	*A630072M18Rik*
IFNβ/γ vs IFNγ	5.06	2.98	2.59	2.33	2.26	2.23	1.49	1.31	1.19	1.17	1.04

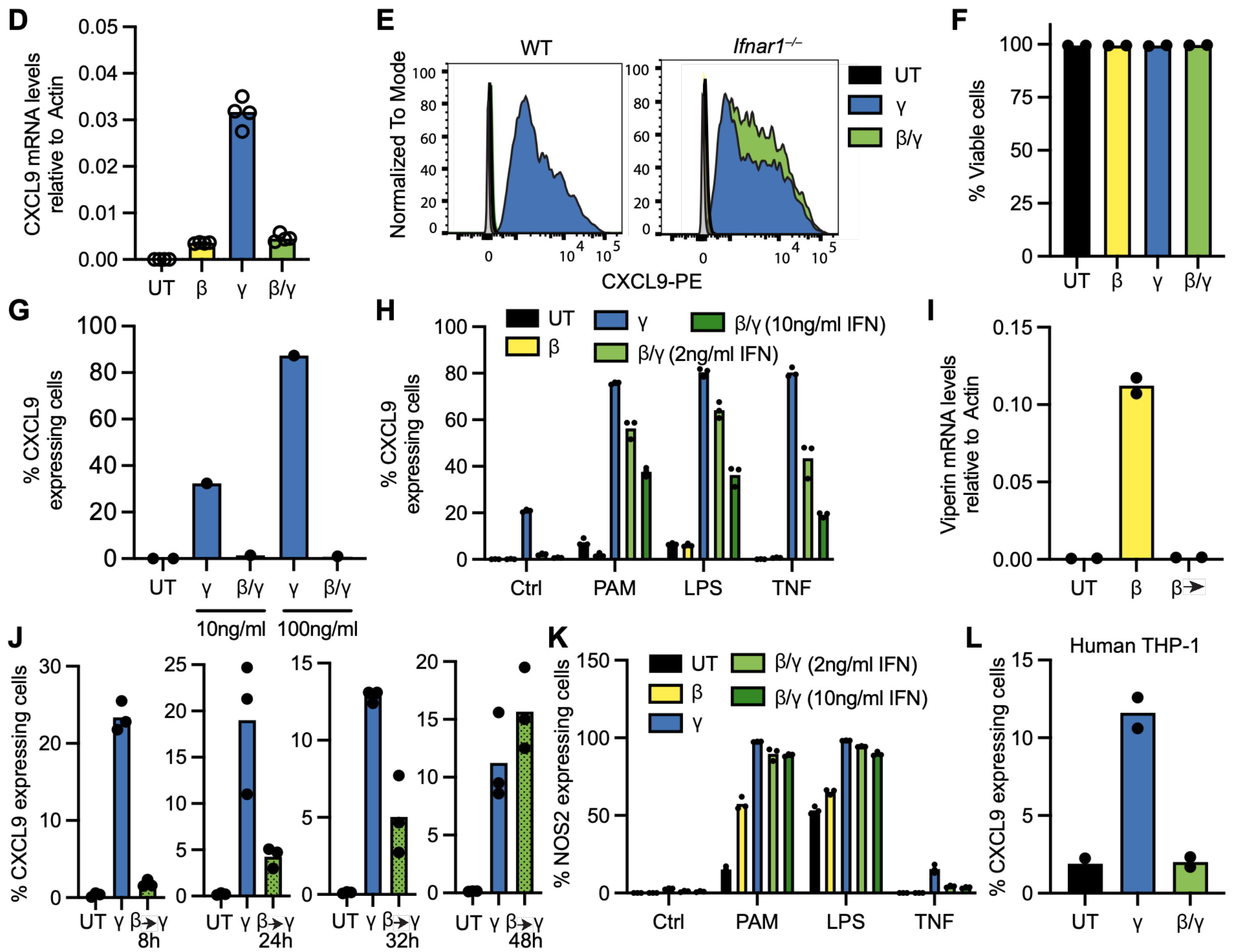

Figure S1. Analysis of type I IFN-driven suppression of IFNγ signaling, related to Figure 1
(**A**) Log$_2$ fold change of IFNγ signature genes in bone marrow derived macrophages (BMMs) upon exposure to IFNγ ± IFNβ pre-exposure (*n* = 3). (**B**) Induction of IFN-specific genes by BMMs upon IFNβ and IFNγ. Genes two-fold more induced upon IFNγ compared to IFNβ are defined as IFNγ specific genes (blue). Average of *n* = 3 is plotted in (B). (**C**) Table of the IFNγ specific genes that are not inhibited by type I IFN signaling. (**D**) Quantification of CXCL9 mRNA levels by RT-qPCR from BMMs exposed to IFNγ ± IFNβ-preexposure. UT = Untreated (**E**) Representative flow cytometry histogram of CXCL9 from WT and *Ifnar1*$^{-/-}$ BMMs exposed to IFNγ ± IFNβ-preexposure. (**F**) Quantification of viable BMMs upon exposure to cytokines. (**G**) Quantification of CXCL9 protein expressing BMMs from WT mice exposed to 10 and 100ng/ml IFNγ ± IFNβ-preexposure. (**H**) Quantification of CXCL9 protein expressing BMMs from WT mice

upon exposure to IFNβ, IFNγ or IFNβ/γ in the absence or presence of tumor necrosis factor (TNF) and Toll-like receptor agonists Pam3CSK4 (PAM) or Lipopolysaccharide (LPS). (**I**) Quantification of *Rsad2* mRNA (encoding Viperin) levels by RT-qPCR of WT BMMs directly upon IFNβ exposure or after removing it for 8h. (**J**) Quantification of CXCL9 protein expressing BMMs from WT mice exposed to IFNγ at various time post transient overnight IFNβ-exposure. (**K**) Quantification of NOS2 protein expressing BMMs from WT mice upon exposure to IFNβ, IFNγ or IFNβ/γ in the absence or presence of TNF and Toll-like receptor agonists PAM or LPS. (**L**) Quantification of CXCL9 protein expressing human THP-1 macrophages exposed to IFNγ ± IFNβ-preexposure. (D, F-L) Representative data from ≥ 3 biological replica with (D, F, H-L) $n \geq 2$ and with (G) $n = 1$ per condition. Statistical significance was calculated in (A) with RM two-way ANOVA with the Geisser-Greenhouse correction and Sidak's multiple comparison test. *$p < 0.05$, **$p < 0.01$, ***$p < 0.001$, ns = not significant.

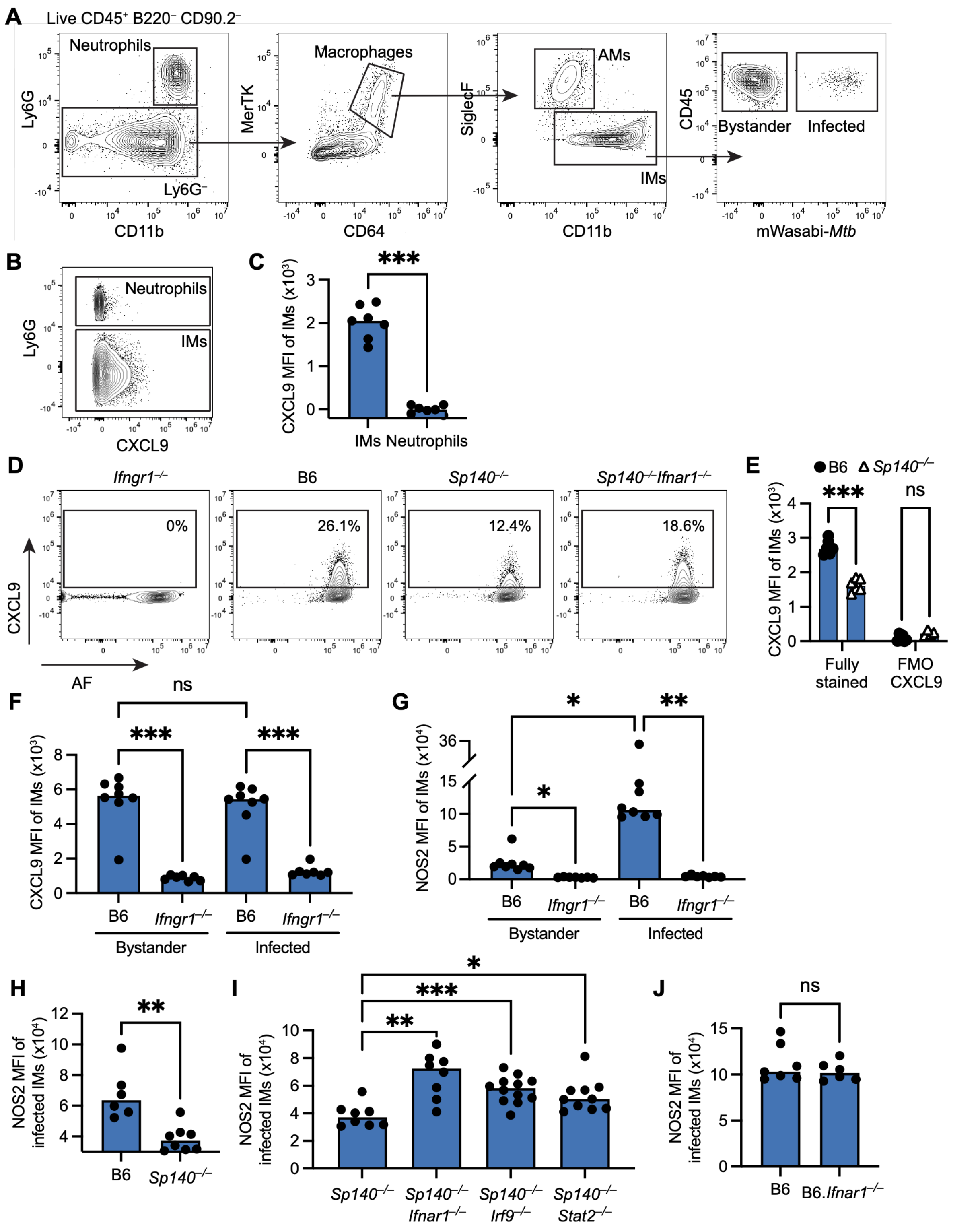

Figure S2. Control data and NOS2 expression in IMs of *Mtb* infected mice, related to Figure 1
(**A**) Representative flow cytometry plots of a *Mtb*-mWasabi infected B6 mouse gated on live CD45⁺ B220⁻ CD90.2⁻ cells to identify interstitial macrophages (CD64⁺ MerTK high SiglecF⁻), which can be grouped into bystander (mWasabi⁻) and infected (mWasabi⁺) cells. (**B**) Representative flow cytometry plots of CXCL9 expression by IMs and neutrophils at day 28-29 pi and in (**C**) corresponding quantification. (**D**)

Representative flow cytometry plots of CXCL9 vs autofluorescence (AF) in indicated mouse strains at day 25-26 pi. (**E**) Quantification of CXCL9 MFI of IMs in fully stained and fully stained minus CXCL9 (FMO CXCL9) samples from *Mtb*-infected B6 and *Sp140*$^{-/-}$ mice at day 25 pi. (**F**) MFI of CXCL9 and (**G**) MFI of NOS2 in IMs from *Mtb*-infected B6 and *Ifngr1*$^{-/-}$ mice at day 25 pi grouped in bystander and infected IMs. (**H**) MFI of NOS2 staining in IMs from *Mtb*-infected B6 and *Sp140*$^{-/-}$ mice at day 25 pi. (**I**) MFI of NOS2 staining in IMs from *Mtb*-infected *Sp140*$^{-/-}$, *Sp140*$^{-/-}$*Ifnar1*$^{-/-}$, *Sp140*$^{-/-}$*Irf9*$^{-/-}$ and *Sp140*$^{-/-}$*Stat2*$^{-/-}$ mice at day 28-29 pi. (**J**) MFI of NOS2 staining in IMs from *Mtb*-infected B6 and B6.*Ifnar1*$^{-/-}$ mice at day 25-26 pi. (C, E, F-J) In each figure, data was combined from ≥ 2 biological replica with n ≥ 6 per group. Statistical significance was calculated in (E-G) with Two-way ANOVA and Sidak's multiple comparison test, in (I) with Brown-Forsythe and Welch ANOVA test and Dunnett's T3 multiple comparisons test, in (C, H, J) with Welch's t-test. *p < 0.05, **p < 0.01, ***p < 0.001, ns = not significant.

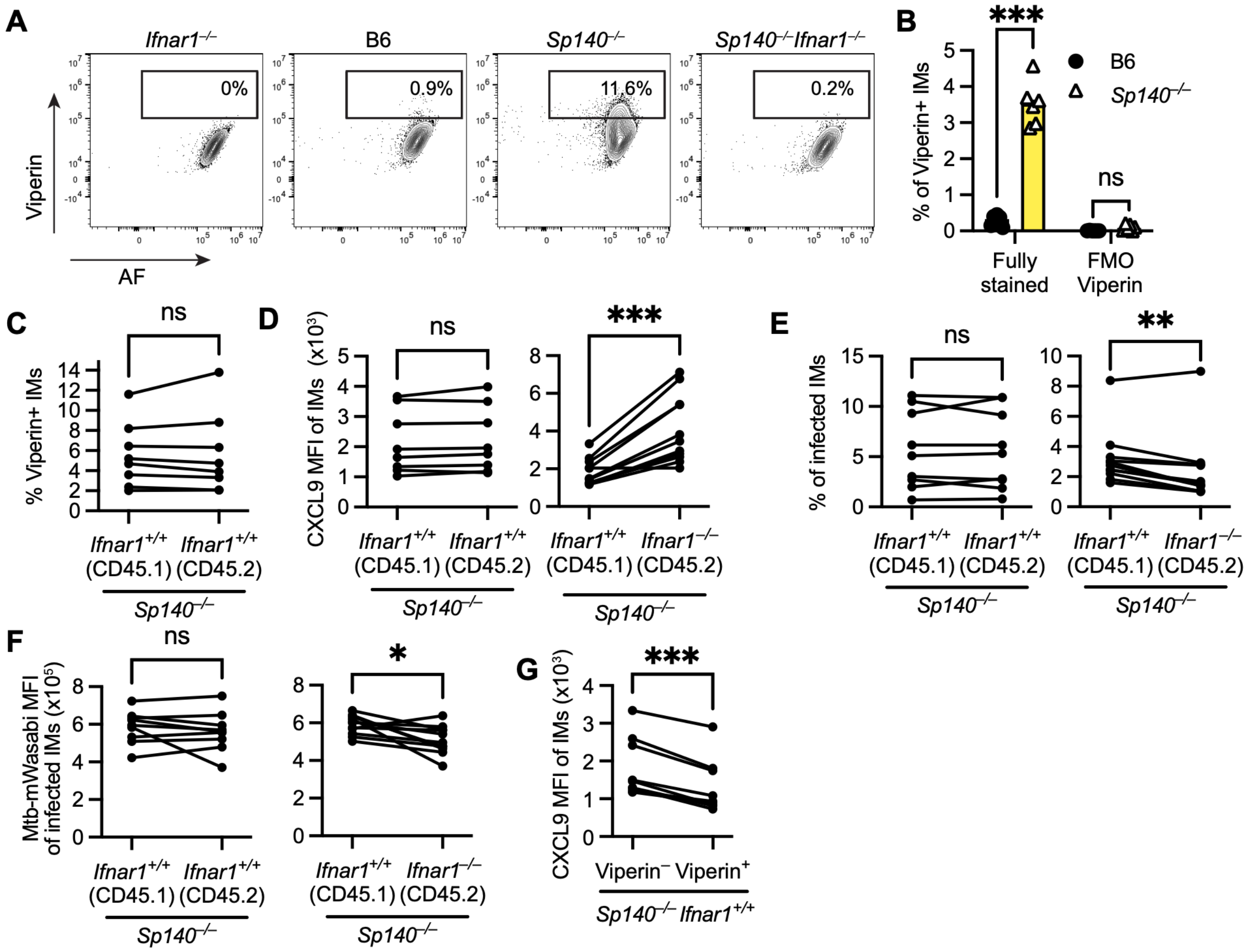

Figure S3. Control data, related to Figure 2

(**A**) Representative flow cytometry plots of Viperin vs autofluorescence (AF) in indicated mouse strains at day 25-26pi. (**B**) Quantification of Viperin MFI of IMs in fully stained and fully stained minus Viperin (FMO Viperin) samples from *Mtb*-infected B6 and $Sp140^{-/-}$ mice at day 25 pi. (**C-F**) Analysis of *Mtb*-infected mixed $Sp140^{-/-}$ bone marrow chimeras containing CD45.1- and CD45.2-positive cells, either both IFNAR-proficient (control) or IFNAR-proficient and IFNAR-deficient, respectively, at day 25-26 pi. Comparison in terms of (**C**) Viperin positivity, (**D**) CXCL9 MFI, (**E**) percent infected IMs and (**F**) *Mtb*-mWasabi MFI. (**G**) CXCL9 MFI analysis of Viperin-negative and -positive IMs from $Sp140^{-/-}Ifnar1^{+/+}$ subpopulation in figure 3J. Data in (B) from one biological replica with n = 5 per group and in (C-G) from ≥ 2 biological replica with n ≥ 6 per group. Statistical significance was calculated in (B) with Two-way ANOVA and Sidak's multiple comparison test, in (C-G) with paired t-test. *p < 0.05, **p < 0.01, ***p < 0.001, ns = not significant.

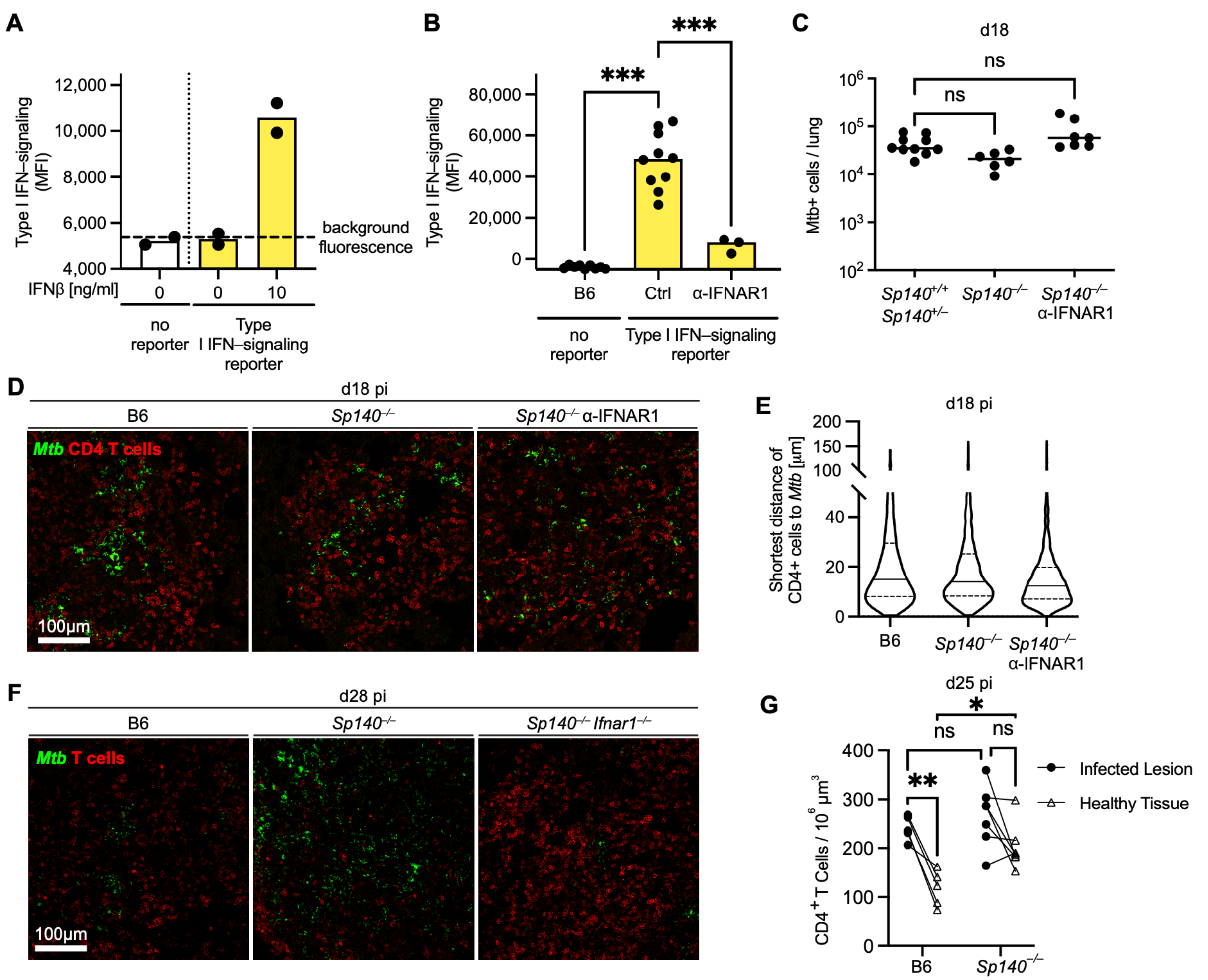

Figure S4. Control data for *Mx1*^{gfp} mouse line and T cell recruitment in B6 vs *Sp140*$^{–/–}$ mice, related to Figure 3

(**A**) Quantification of Mx1-expression of BMMs established from type I IFN-signaling reporter mouse line (*Mx1*^{gfp}) upon exposure to IFNβ compared to background fluorescence. (**B**) MFI of Mx1-GFP expression in IMs from *Mtb*-infected *Mx1*^{gfp} mice without or with administration of anti-IFNAR1 antibodies. (**C**) Quantification of *Mtb*-mCherry-positive cells from *Mtb*-infected *Sp140*$^{+/+}$ *or Sp140*$^{+/–}$ and *Sp140*$^{–/–}$ mice without or with administration of anti-IFNAR1 antibodies at day 18 pi. (**D**) Representative micrograph with CD4^{+} T cells of infected lung lesions from *Mtb*-infected B6, *Sp140*$^{–/–}$ and *Sp140*$^{–/–}$ treated with anti-IFNAR1 antibodies at day 18 pi. (**E**) Shortes distance quantification of CD4^{+} T cells to *Mtb* within infected lesions at day 18 pi. (**F**) Representative micrograph with T cells of lung lesions from *Mtb*-infected B6, *Sp140*$^{–/–}$ and *Sp140*$^{–/–}$ treated with anti-IFNAR1 antibodies at day 28 pi. (**G**) Quantification of CD4^{+} T cells per 10^6 μm^3 in "Infected Lesions" and "Healthy Tissue" from B6 and *Sp140*$^{–/–}$ *Mtb*-infected mouse lungs at day 25 pi. Data in (A) from one biological replica with n = 2 per group and in (B, C, E, G) from ≥ 2 biological replica with n ≥ 3 per group. Statistical significance was calculated in (B) with Brown-Forsythe/Welch ANOVA test and Dunnett's T3 multiple comparisons test, in (C) with Kruskal-Wallis test and Dunn's multiple comparison test, in (G) with an ordinary two-way ANOVA and Turkey's multiple comparisons test. *$p < 0.05$, **$p < 0.01$, ***$p < 0.001$, ns = not significant.

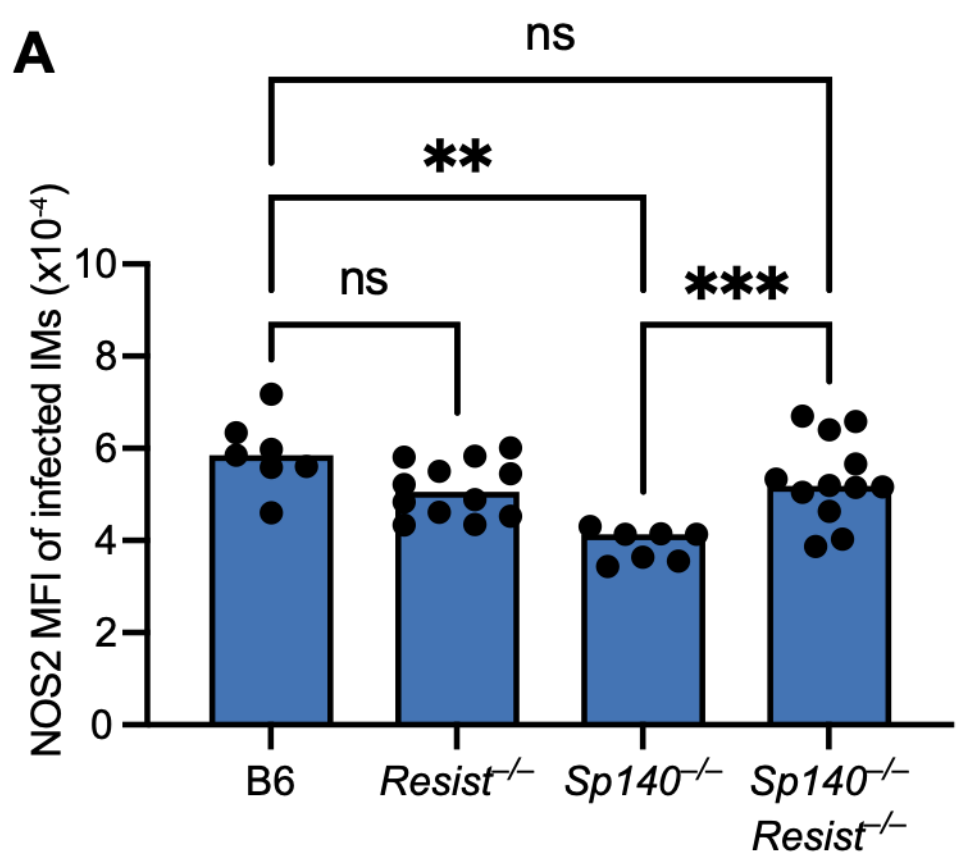

Figure S5. NOS2 expression in *Resist*-deficient mice, related to Figure 4
(**A**) MFI of NOS2 staining in IMs from *Mtb*-infected B6, *Resist*$^{-/-}$, *Sp140*$^{-/-}$, *Sp140*$^{-/-}$*Resist*$^{-/-}$ mice at day 26-28 pi. Statistical significance was calculated with Brown-Forsythe/Welch ANOVA test and Dunnett's T3 multiple comparisons test. *p < 0.05, **p < 0.01, ***p < 0.001, ns = not significant.
